# Quantifying Dissemination of Antibiotic Resistance Genes in Air from a Dairy Farm and Swine Farm

**DOI:** 10.1021/acsestair.5c00055

**Published:** 2025-07-18

**Authors:** David A. Kormos, Gabriel Isaacman-VanWertz, Jactone A. Ogejo, Amy Pruden, Linsey C. Marr

**Affiliations:** † Department of Civil and Environmental Engineering, 543318 Virginia Tech, Blacksburg, Virginia 24061, United States; ‡ Department of Biological Systems Engineering, Virginia Tech, Blacksburg, Virginia 24061, United States

**Keywords:** bioaerosol, conditional sampler, antimicrobial resistance (AMR), animal feeding operation

## Abstract

Farms are a suspected source of dissemination of antibiotic resistance genes (ARGs) to the atmosphere, but their contribution remains poorly quantified. This study investigated the concentrations, emission rates, and particle size distributions of ARGs in air around a dairy farm and swine farm, as well as in farm wastewater and soil as potential sources, during a yearlong sampling campaign. Analysis targeted genes corresponding to a cross-section of antibiotic classes used in human and veterinary medicine, along with 16S rRNA and *intI1* as indicators of total bacterial load and anthropogenic sources of ARGs, respectively. Two approaches were demonstrated for estimating emissions to account for the physical configurations of the farms. A custom sampler that collected size-resolved aerosol particles at a flow rate of 2.25 L/min only when the wind originated from the direction of interest was used to collect aerosol particles near potential sources. At the dairy and swine farms, *bla*_*CTX‑M1*_ concentrations varied significantly by sampling location, averaging 10^2^ gene copies per cubic meter (gc m^–3^) across seasons and peaking at 10^4^ gc m^–3^ during the summer sampling period. At the swine farm, maximum concentrations reached 10^5^ gc m^–3^ for *intI1*, *ermF*, and *qnrA* near the buildings’ exhaust fans. Emission rates reached ∼ 10^5^ gc s^–1^ for some ARGs, including *bla*_*CTX‑M1*_, and 10^6^ gc s^–1^ for *intI1*. ARGs were predominantly associated with coarse particles (>5 μm) near emission sources and were also present in fine (<1 μm) and accumulation (1–5 μm) mode particles near the source and at downwind locations, indicating potential for inhalation exposure and long-range transport.

## Graphical abstract

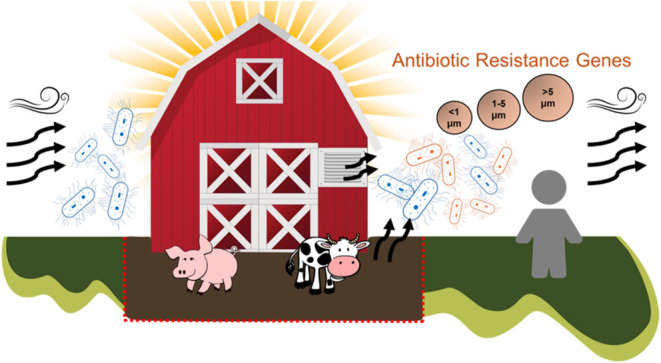

## Introduction

Antimicrobial resistance (AMR) is a major threat to public health and is a serious global concern.^1,2^ Antibiotic resistance genes (ARGs) encode the ability to resist antibiotic treatments and can spread among bacteria, resulting in failure of antibiotics to cure life-threatening infections. ARGs in the atmosphere, carried in aerosol particles, are especially concerning, due to their potential for long-distance transport of AMR.^3^ Studies of ARGs in the atmosphere have been reported in recent years, but significant knowledge gaps remain. For instance, the magnitude of ARG emissions to the atmosphere from specific sources and the size of the carrier particles remain largely unquantified.

Existing studies of ARGs in air have focused on urban areas, rural areas, indoor air, or outdoor air near specific sources, such as hospitals, WWTPs, landfills, or industrial sites.^3–5^ Most studies have used conventional sampling methods to collect total suspended particles, coarse particulate matter 10 μm and smaller (PM_10_), fine particulate matter 2.5 μm and smaller (PM_2.5_), or other size fractions. Analytical methods for detecting ARGs include polymerase chain reaction (PCR), quantitative PCR (qPCR), droplet digital PCR (ddPCR), and metagenomics. Among PCR-based methods, specific genes that encode resistance to various classes of antibiotics (sulfonamides, tetracyclines, β-lactams, glycopeptides, macrolides, quinolones) as well as mobile genetic elements (MGEs) have been targeted. Often, studies also report ARGs in terms of relative abundance, which is normalized to a measure of the size of the total bacterial population. Relative abundance is a useful indicator of the proportion of the microbial community carrying ARGs, but it does not translate well toward quantifying ARG fate, transport, or exposure in air, given that bacterial population sizes can vary widely in airborne matrices. For example, relative abundance could be low in samples collected in a dust storm carrying a high load of bacteria together with ARGs.^6,7^

Agricultural activity in rural settings has been well-established to be a major driver of the dissemination of ARGs in the environment.^5^ However, few studies demonstrating the transport of ARGs in the air have been conducted at rural sites near farms.^8–10^ Animal feces and soil are suspected sources for airborne ARGs at animal farms, as they have been shown to be for neighboring waterways,^11,12^ indicated by similarity in ARG profiles.^5^ ARG profiles in air have been found to differ by farm, affected by the livestock, farming practices, and antibiotic usage patterns, as well as surrounding areas including other farms or towns.^13–16^ A study at a swine farm in Guizhou, China indicated the possibility of long-range airborne transport of ARGs, as samples from some farms were abundant in the ARG aminocoumarin even though they did not use the corresponding class of antibiotics. This class, which includes novobiocin, is used for boars at other locations in China. Another study found increased levels of the colistin resistance gene (*mcr-1*) in airborne dust in Eastern Canada, even though the surrounding area had imposed restrictions on the use of colistin.^17^

Many questions remain about the occurrence patterns and health implications of ARGs in the air near farms. The purpose of this study was to quantify the distribution of ARGs representing a cross section of key antibiotic classes used in human and veterinary medicine (tetracyclines, quinolones, macrolides, glycopeptides, and β-lactams) and the *intI1* class I integron integrase gene, an MGE^12^ that is commonly used as an indicator of mobile, anthropogenic sources of AMR, on two distinct farm types (swine and dairy) in the same region. To achieve this purpose, we developed and demonstrated approaches for quantifying emissions of ARGs from agricultural sources, which allowed us to characterize potential sources (farm wastewater and soil) of ARGs found in the air, the corresponding particle size distribution, and the potential for downwind transport. If ARGs are present mainly in larger, coarse particles, then long-distance transport is less of a concern because these particles would settle to the ground rapidly, and vice versa. We developed a custom air sampler that collected size-resolved aerosol particles only when the wind originated from the direction of a designated source of interest. This enabled measurement of the size distributions and emission rates of ARGs from sources of interest. We also evaluated relationships among specific ARGs, environmental factors, and gene co-occurrence to gain insight into drivers of observed ARG patterns.

## Methods

The Virginia Tech Dairy Science Complex at Kentland Farm is a state-of-the-art research and teaching facility in Blacksburg, Virginia (Figure S1). The farm maintains a diverse herd of approximately 250 Holstein and Jersey cows and heifers and calves. The complex includes advanced milking systems, housing designed for animal comfort, and laboratories for data collection and analysis. The open barns are naturally ventilated with ceiling fans, to enhance cooling, set to turn on at 14 °C for the cows' comfort. The thermostat-controlled ceiling fans in the special-needs barn are angled downward and slightly in the downwind direction. The facility also features a special-needs and heifer barn with a capacity for 50 cows and 40 heifers, supporting targeted research and care. The waste management system incorporates hydraulic flushing, sand bedding and recovery, and a weeping wall for solids collection. The separated liquids from the weeping wall are stored in two large in-ground concrete tanks, each with a nominal capacity of 9 million liters. The liquid for hydraulic flushing is obtained from the manure storage tanks.

The Virginia Tech Swine Farm is a research facility with a herd of pigs raised in mechanically ventilated buildings (Figure S1). Used for various research and educational purposes, the facility includes areas for housing up to ∼100 pigs at different stages of growth, from farrow to finish. The dairy and swine farms mainly use β-lactam antibiotics to treat their animals (ceftiofur and penicillin), although there is also a recent history of tetracycline use (chlortetracycline) at these facilities, as well as macrolide (tylosin) use at other animal operations run by the university in close proximity (∼1 km) to the swine farm.

### Collection and Preparation of Air Samples

Sampling was conducted at each farm over 13 months, in 2022–2023, on 4–5 days during each season. For the purposes of this analysis, the sampling days during each season are grouped into a single event, with four events over the entire study. Different aerosol collection approaches were applied to the dairy and swine farms due to their distinct layouts, aligning with the goal of demonstrating an adaptive approach to estimating emissions from various agricultural sources. The dairy farm had open-air barns surrounded by grazing land, while the swine farm comprised several buildings with mechanical ventilation, controlled by a thermostat, and was situated close to other campus and agricultural facilities. At the dairy farm, sampling was conducted along a transect downwind of the special-needs barn on days with similar wind speed and direction. Sampling periods were selected by monitoring weather forecasts for days with wind speed greater than a light breeze (1.5 m s^–1^) in the desired direction. At the swine farm, sampling was conducted on days with low wind speed (less than 0.75 m s^–1^) so that samples collected near the building’s exhaust fans would be dominated by the exhaust and not outdoor air. Size-segregated particle samples were collected for 24 h at a time using a cascade impactor (SKC Sioutas Personal Cascade Impactor, Eighty Four, PA) at a flow rate of 2.25 L min^–1^, resulting in cutpoints of 5, 2, 1, and 0.5 μm. Particles deposited on sterilized PTFE filters (SKC, Eighty Four, PA) on each impactor stage and an after-filter to collect particles smaller than 0.5 μm.

“Conditional” air samplers were designed and built to collect particles under certain, predefined conditions, namely when the wind speed and wind direction met specific criteria. The sampler package consisted of an impactor, microcontroller (PJRC Teensy 3.5, Sherwood, OR), micro diaphragm pump (Parker Hannifan T2–03, Mayfield Heights, OH), flow meter (Honeywell Zephyr HAFUHT0010L4AXT, Charlotte, NC), and battery (Voltaic Systems V50, Brooklyn, NY). The microcontroller monitored the analog signal from an attached wind anemometer and vane (Davis Instruments 6410, Hayward, CA). All components operated at 5 V using a standard USB power supply. A step-up convertor (SparkFun COM-15208, Niwot, Colorado) was used to provide 6 V to the pump, which was switched on and off using an electromagnetic relay (DaFuRui DC-08, Shenzhen, China). The microcontroller recorded and stored measured flow rate and wind data to an onboard SD card with 16-bit analog-to-digital conversion. All electronics were housed in a weatherproof box, with wind instruments mounted externally, constituting a relatively portable measurement and sampling package. The inside of the sampler package is shown in Figure S2. Temperature and relative humidity were recorded using a HOBO logger attached to one of the samplers, and regional PM_2.5_ data was collected from a Purple Air monitor located on the main campus of Virginia Tech, ∼15 km from the dairy farm and ∼3 km from the swine farm.

At the dairy farm, one conditional sampler was placed upwind of the special-needs barn, and three were placed at 5, 65, and 115 m downwind (Figure S1). The samplers were programmed to turn on when the wind speed exceeded 0.1 m s^–1^ and the wind direction was within 160 degrees of the special-needs barn at 5 m downwind and within 120 degrees from the special-needs barn at 65 and 115 m downwind and turn off when conditions were not met. At the swine farm, a sampler was placed near a road, 50 m away from the buildings, and two samplers were placed near the buildings’ exhaust fans (Figure S1), 1 m away at a height of 1.5 m. These were referred to as “exhaust fan 1” for the main building, which houses farrowing, nursing, and some finishing pigs, and “exhaust fan 2” for the smaller building that houses grower pigs, which were in the growth phase between weaning and finishing. Pigs were in their most vulnerable stage for sickness, and most likely to receive antibiotics when they were first weaned and moved to the nursery, and during the farrowing stage. Conditional sampling was not used at the swine farm. Instead, sampling was conducted on days with still or light winds (less than 0.75 m s^–1^) blowing from the road toward the buildings, allowing the road to serve as the background site.

### Collection of Farm Wastewater, Soil, and Dust Samples

Due to the potential for aerosolization from nearby liquid and solid sources to contribute to AMR in the atmosphere, wastewater (separated liquid manure) samples were collected from the manure storage tanks adjacent to the dairy barn at the time of air sampling (Figure S1), and soil samples for both farms were collected from the topmost layer of the ground. Wastewater samples were concentrated onto 0.22 μm pore size, 47 mm diameter filters (Millipore Sigma, Burlington, MA) and the corresponding filtered volume recorded. Following soil testing guidelines,^18^ approximately 1 g of soil was collected at four points around each sampler on each sampling day. These soil samples were then mixed and combined into one vial to create a composite sample for each day (generally 12 g at the swine farm, and 16 g at the dairy farm). Additionally, manure from the special-needs barn was collected into a separate vial utilizing a similar method at four different points within the pens in the barn, and dust from the ventilation exhaust fan housing at the swine farm was collected into a separate vial on each day of sampling.

### Quantification of ARGs and Class 1 Integrons

DNA was extracted from all samples using the FastDNA Spin Kit for Soil.^19^ Filters containing concentrated air or wastewater samples, or approximately 0.5 g of soil or dust, were added directly to the extraction tubes. DNA extracts were analyzed in triplicate using the CFX96 Touch Real-Time PCR Detection System (BioRad Laboratories, Hercules, CA) using SsoFast Evagreen Supermix (BioRad Laboratories, Hercules, CA) and then, in the case of air samples, converted to gene copies per cubic meter of air (gc m^–3^) by dividing by the volume of air sampled. The ARG targets were *qnrA*,^20^ *ermF*,^21^ *sul1*,^12^ *tetA*,^12^ *vanA*,^12^ and *bla*_*CTX‑M1*_ ^12^ in air, soil/dust, and wastewater samples. Additionally, the *intI1* class 1 integrase-specific gene^22^ and total bacterial 16S rRNA genes^23^ were also measured. Protocols and primers for each gene are listed in Table S1.

ARGs and the MGE were selected to represent resistance to a cross-section of antibiotic classes used in human and veterinary medicine. Further, *intI1* and *sul1* serve as indicators of anthropogenic sources of AMR, with *intI1* also indicating active horizontal gene transfer processes.^12,24,25^ The farms sampled in this study both used β lactam antibiotics as a last line of defense for treating sick animals, specifically Spectramast LC (ceftiofur, cows), Excede (ceftiofur, weaning and nursing pigs), and penicillin G procaine (farrowing pigs). Additionally, Quartermaster, which includes dihydrostreptomycin sulfate, an aminoglycocide, and penicillin G procaine, was used on the dairy farm for more severe or persistent infections if ceftiofur or other dry cow therapies failed. Although the exact frequency of antibiotic use throughout the sampling year was unknown, anecdotal evidence and manure testing suggested that antibiotics were used several times each season for sick cows and pigs in the sick pen and pig facilities. A previous study at the Virginia Tech Dairy Complex reported that ceftiofur administration in dairy cows resulted in elevated levels of β-lactam resistance genes in their feces,^10^ further supported by other qPCR studies of similar environments.^26–28^ *bla*_*CTX‑M1*_ was also a target of interest for this study because it encodes resistance to β lactam antibiotics used on the farms and because it is often carried by resistant human pathogens. qPCR was the method of choice because it is more sensitive than metagenomic analysis, which does not consistently capture effects of changes in antibiotic usage patterns of less abundant genes like *bla*_*CTX‑M1*_.^27–29^ *tet*A was of interest as it encodes resistance to tetracyclines, which are commonly used in livestock, and while chlortetracycline use has been linked to increased levels of *tetA*, so has use of nontetracyclines, such as ceftiofur.^27–29^ The swine farm had previously used chlortetracycline to treat their pigs, and some facilities within 1 km had previously used macrolides, resistance to which is encoded by *erm*F, to treat their animals (sheep and horses). While corresponding antibiotics were not directly used to our knowledge during the period of this study, *tetA* and *ermF* hypothetically could still affect the swine farm via transport of aerosols from surrounding areas, including these other farms and historical usage.^13–16^ Despite their associated antibiotics not being used at these locations, *qnrA* and *vanA* were included to represent two prominent ARG classes and because they are both clinically relevant genes in agriculture.

### Estimation of Emission Rates

Emission rates were estimated and reported as gene copies per second. For the dairy farm, an inverse Gaussian dispersion modeling approach was used to estimate the emission rate from concentrations measured downwind. This approach approximates the source as a point source and has been used to model dispersion and deposition of fungal spores from a ryegrass field and aerosols generated by compost facilities and land application of manure.^30–33^ Briefly, because the relationship between the concentration and emission rate is linear and the decrease in concentration as a function of distance is constrained by the model parameters, one can fit an emission rate that best predicts the observed concentrations.^33^ The model does not account for particle deposition, so the estimates represent emissions of smaller particles and not coarse ones that are rapidly removed by settling. Further details on the Gaussian dispersion model implemented can be found in the Supporting Information (Supporting Information, Text S1).

Measurements of gene concentrations from the sites at 65 and 115 m were used, while measurements from the site at 5 m were excluded because it was too close to the source, limiting the robustness of the regression analysis. The ratio was calculated by first subtracting the background (upwind) concentration from those measured at the two downwind locations, plotting the observed downwind concentrations against concentrations predicted with a unit emission rate, and calculating the emission rate as the slope between observed and predicted concentrations. Modeling more than 1 day for each site provided a measure of day-to-day variability. There were several assumptions inherent to this method. Because the concentration measurement was time-integrated, the derived emission rate represented a time-integrated value and did not capture short-term temporal variability in source strength or wind speed. Neutral conditions, Pasquill stability class D, were assumed as a compromise between stable and unstable conditions that typically occur over a 24 h period. The topography around the sites was flat, so the model did not need to account for complex terrain.

The emission rate at the swine farm was calculated as the product of the exhaust fan’s volume flow rate and the background-subtracted concentration of ARGs or other measures of AMR. The volume flow rate was estimated by measuring air velocity in the exhaust duct and multiplying it by the cross-sectional area.

### Statistical Analysis

Analyses were performed in R employing the Kruskal–Wallis test to determine differences in bacterial load across the sampling events, locations of the samplers, and size fraction of the samples. If significant effects were found, post hoc tests were applied to assess conditions under which the bacterial load varied significantly. The Kruskal–Wallis test was conducted with a 95% confidence interval, and the Dunn’s post hoc tests were adjusted for multiple comparisons using the Bonferroni correction to control for Type I error. Spearman correlation coefficients were calculated to compare targeted gene measurements in air with measurements in potential environmental sources and with environmental conditions, and to assess co-occurrence among the gene targets. The threshold for significance was a p-value of 0.05. Figures were generated with R Studio.

## Results

### ARG Concentrations in Air at the Dairy Farm

Measurements of targeted gene concentrations were evaluated as independent samples per sampling event (one per season), sampling site, and sampling day, further subdivided into five size fractions from the impactor. These independent samples of targeted gene concentrations from the size-segregated impactor for each independent sample were then summed together to create a total aerosol (i.e., not size-segregated) concentration and grouped by sampling event or site to estimate the average concentration of each gene target (Figure 1). Relative abundances were calculated as the proportion of gene copies of the target to those of the 16S rRNA gene representing total bacteria, (Figure S3). Spatial differences in ARG occurrence across the dairy farm locations were apparent, with the highest concentrations generally observed in the downwind samples closest to the barn. Further, the total sum of all concentrations of ARG targets and *intI1* was significantly highest during the summer sampling event, likely driven by *intI1* trends, while the lowest total concentration was recorded at the upwind site in winter (Figure S4). Upwind and 115 m downwind concentrations were not significantly different for any gene.

**Figure 1:**
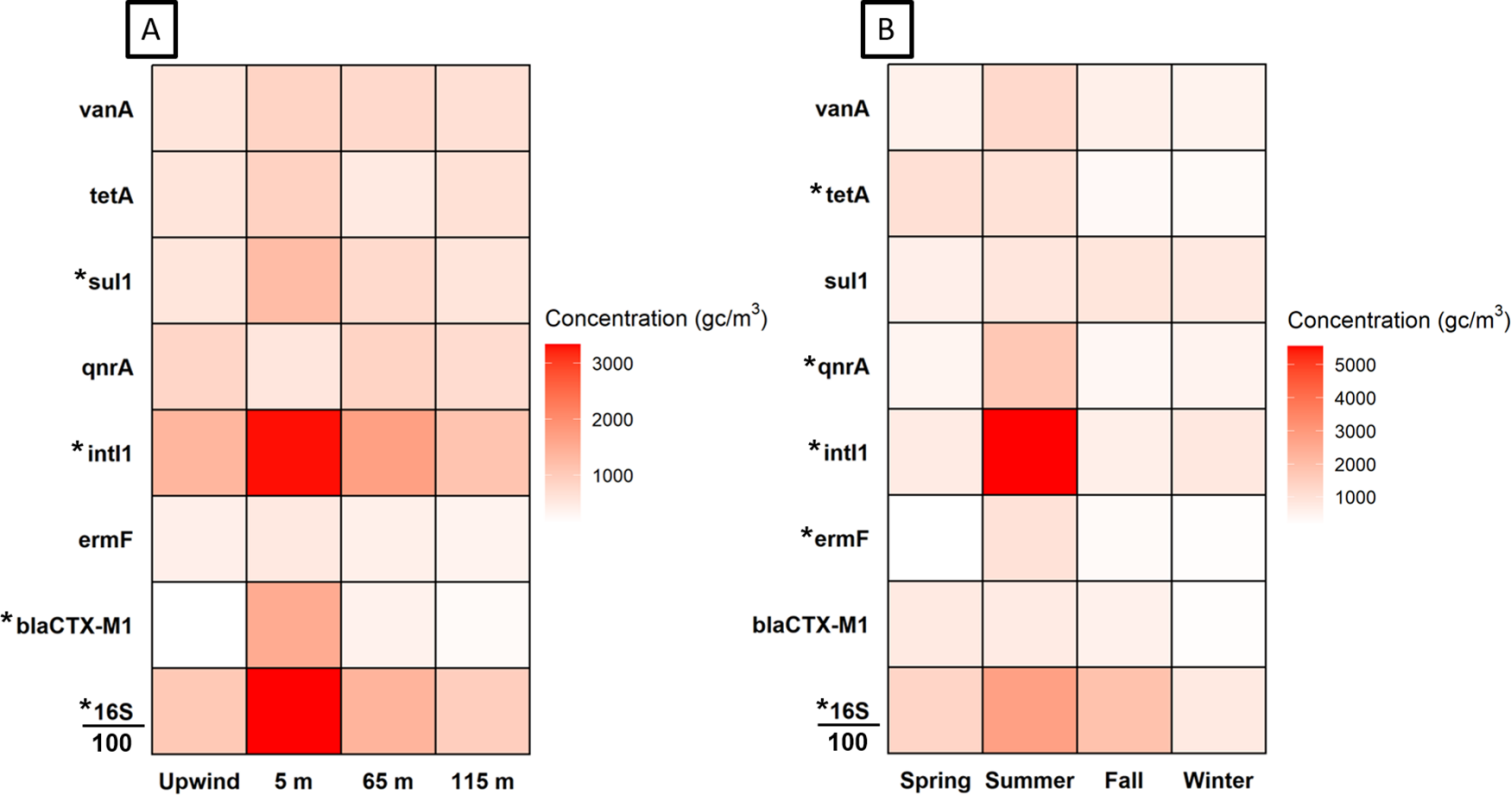
Heat map of average concentrations of each targeted gene measured in air samples at the dairy farm, (A) by distance downwind of the special-needs barn and (B) by season. * indicates significant difference by season or distance from Kruskal–Wallis and Dunn’s posthoc tests. 16S rRNA gene concentrations indicative of total bacterial populations were divided by 100 to aid comparison on the same scale.

Spatially, *tetA*, *sul1*, *intI1*, *bla*_*CTX‑M1*_, and bacterial 16S rRNA gene concentrations were elevated at the 5 m location compared to other sites (Figure 1A). Specifically, *bla*_*CTX‑M1*_ was significantly higher at 5 m than at 115 m downwind and upwind, *intI1* was significantly higher at 5 m than upwind, and *sul1* was significantly higher at 5 m than at 115 m. A clear downwind transport pattern was evident from the special-needs barn for *bla*_*CTX‑M1*_ and *intI1* across all events (Figure S4) and for *sul1* and *ermF* during the summer event. No consistent spatial trend was noted for *qnrA* or *vanA*, although concentrations of both were elevated at all sampling sites throughout the year.

Temporally, *intI1* stood out with the highest concentration during the summer sampling days, on the order of 10^4^ gc m^–3^, and concentrations of bacterial 16S rRNA genes were also elevated at this time (Figure 1B). Concentrations of *ermF*, *qnrA*, and *tetA* were also significantly higher during the summer sampling event, with maximum concentrations on the order of 10^3^ gc m^–3^, and averages on the order of 10^2^ gc m^–3^. Concentrations of *bla*_*CTX‑M1*_ and *vanA* were similar in magnitude and did not vary significantly across sampling events. While the maximum *sul1* concentration was observed during the winter, there was no statistical difference across the sampling events. During the summer event, *intI1* concentrations were significantly higher at both the 5 and 65 m locations compared to the other events (Figure S4).

### ARG Concentrations in Air at the Swine Farm

Sampling at the swine farm exhaust fans provided a means to directly measure emitted gene concentrations, which were found to be associated with fan run time (Table S2). Relative abundances for the swine farm are shown in the SI (Figure S5). At exhaust fan 1, the highest concentrations of ARGs were observed when run times exceeded 20 h. For all genes except *vanA*, concentrations were significantly lowest when the fan ran for less than 5 h. Concentrations of *intI1, qnrA*, and *vanA* reached a maximum of 10^5^ gc m^–3^, whereas concentrations of *bla*_*CTX‑M1*_, *ermF*, *sul1*, and *tetA* peaked around 10^3^ to 10^4^ gc m^–3^. Run time was also a significant factor at exhaust fan 2 (Table S3), where targeted gene concentrations were generally lower than at exhaust fan 1 (in 10/13 samples collected when both fans were running). *bla*_*CTX‑M1*_, *intI1*, *qnrA*, *ermF*, and *tetA* all increased with increased run times. The road site (Table S4) showed significantly lower concentrations of all targeted genes compared to exhaust fan 1, and significantly lower concentrations of *bla*_*CTX‑M1*_, 16S rRNA, and *van*A compared to exhaust fan 2. Concentrations at the road site were not correlated with exhaust fan run times. Most of the maxima for each ARG occurred on the same sampling day during the summer event, when the exhaust fan ran for the full 24 h and the number of pigs present was the second highest observed, 83.

### Associations with Potential Environmental Sources and Conditions

Some correlations were found between ARG concentrations in the air and corresponding environmental conditions or potential sources. At all sites at the dairy farm, wind speed was either not correlated or negatively correlated with ARG concentrations, consistent with higher wind speeds enhancing dispersion and dilution of airborne particles emanating from the farm.^34^ Upwind of the special-needs barn, PM_2.5_ and temperature were the factors most strongly correlated with concentrations for all genes, except for *bla*_*CTX‑M1*_ and *sul1* (Figure 2A). *ermF* was the only gene whose concentration in air was significantly correlated with wastewater concentration.

**Figure 2:**
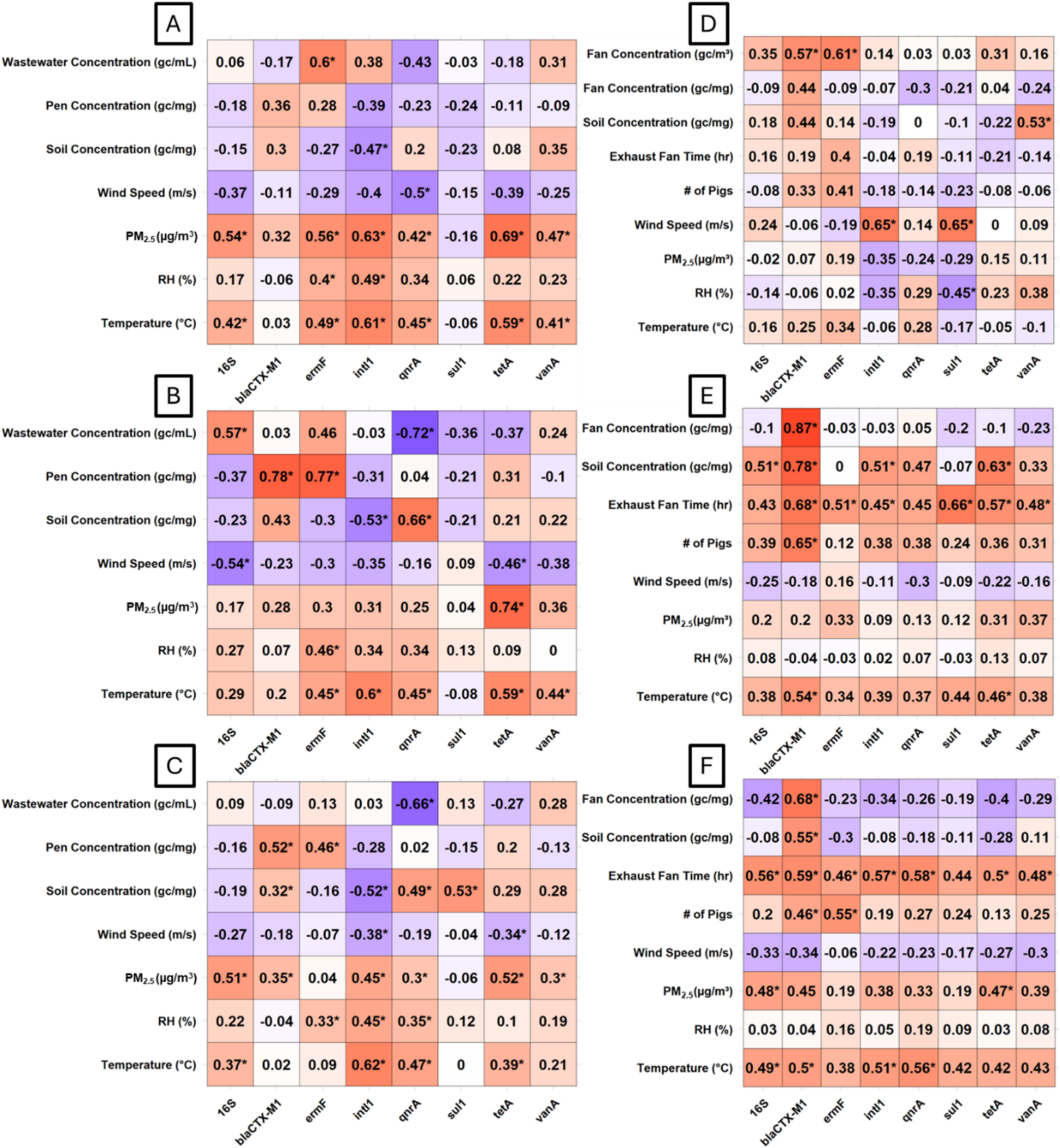
Spearman correlation coefficients between airborne bacteria, ARG, and MGE concentrations along the *x*-axis, with concentrations of these targets in nearby wastewater, pen manure at the dairy farm, or dust on the fan housing at the swine barn, soil, and environmental conditions including wind speed, regional particulate pollution as PM_2.5_, relative humidity (RH), and temperature along the *y*-axis. Target genes measured at the dairy farm (A) upwind, (B) 5 m downwind, and (C) combined downwind sites at 65 and 115 m, and measured at the swine farm at (D) road, where the two fan concentrations refer to aerosols at the fans in gc/m^3^ and dust on the fan housing in gc/mg, (E) exhaust fan 1, and (F) exhaust fan 2 sites. Size-segregated gene concentrations were summed for each independent sampling day, with each correlogram representing 15–20 samples collected over the course of a year at each site. Environmental conditions were averaged over a 24 h period for that sampling day.

At 5 m downwind of the special-needs barn (Figure 2B), airborne concentrations of *bla*_*CTX‑M1*_ and *ermF* were significantly correlated with those in manure gathered from the pen. Temperature and, by association, the ceiling fan run time were significantly correlated with concentrations of *intI1*, *ermF*, *qnrA*, and *tetA*. The relationship at the upwind location was similar, which suggests that temperature rather than the fan was the more relevant factor in aerosol concentrations, as the fan would not have affected the upwind site. *qnrA* was the only gene whose concentrations were significantly correlated with those in soil. We combined observations at 65 and 115 m for downwind analysis, as no significant differences were found between these sites according to the Kruskal–Wallis test. At the downwind sites (Figure 2C), *bla*_*CTX‑M1*_ and *ermF* remained significantly correlated with pen manure concentration, while *intI1*, *qnrA*, and *tetA* were still significantly correlated with temperature and PM_2.5_. Additionally, *qnrA* and *sul1* were significantly correlated with soil concentration.

At the swine farm, ARG concentrations at the exhaust fans were most strongly correlated with exhaust fan run time and were further related to temperature and the number of pigs present. At the road site (Figure 2D), aerosol concentrations of *bla*_*CTX‑M1*_ and *ermF* were correlated with those at the exhaust fans. Concentrations of *sul1* and *intI1* were significantly positively correlated with wind speed, indicating that there may be substantial upwind sources of these common anthropogenic ARGs. At exhaust fan 1, run time was significantly correlated with concentrations of all genes, except *qnrA* and 16S rRNA (Figure 2E), while the number of pigs was only significantly correlated with *bla*_*CTX‑M1*_. At exhaust fan 2 (Figure 2F), fan time was significantly correlated with concentrations of *bla*_*CTX‑M1*_, *intI1*, *qnrA*, *ermF*, *vanA*, and *tetA*, while the number of pigs was significantly correlated with concentrations of *ermF* and *bla*_*CTX‑M1*_. Aerosol and fan housing dust concentrations were significantly correlated for *bla*_*CTX‑M1*_, although the relationship was not as strong as was observed exhaust fan 1. Soil and aerosol concentrations were significantly correlated only for *bla*_*CTX‑M1*_ at the swine farm.

### Co-Occurrence Patterns Among ARGs

At the upwind site, *ermF*, *qnrA*, *tetA*, and *vanA* were significantly correlated with *intI1* and with each other (Figure 3A). *blaCTX-M1* showed no significant association with any other genes. At the 5 m location, only *qnrA* and *vanA* remained significantly correlated with *intI1*, while *bla*_*CTX‑M1*_, *ermF*, and *tetA* were not strongly correlated with other genes (Figure 3B). At the downwind locations, *qnrA* and *vanA* were significantly correlated with *intI1*. No other significant co-occurrence patterns were observed at the downwind sites (Figure 3C).

**Figure 3:**
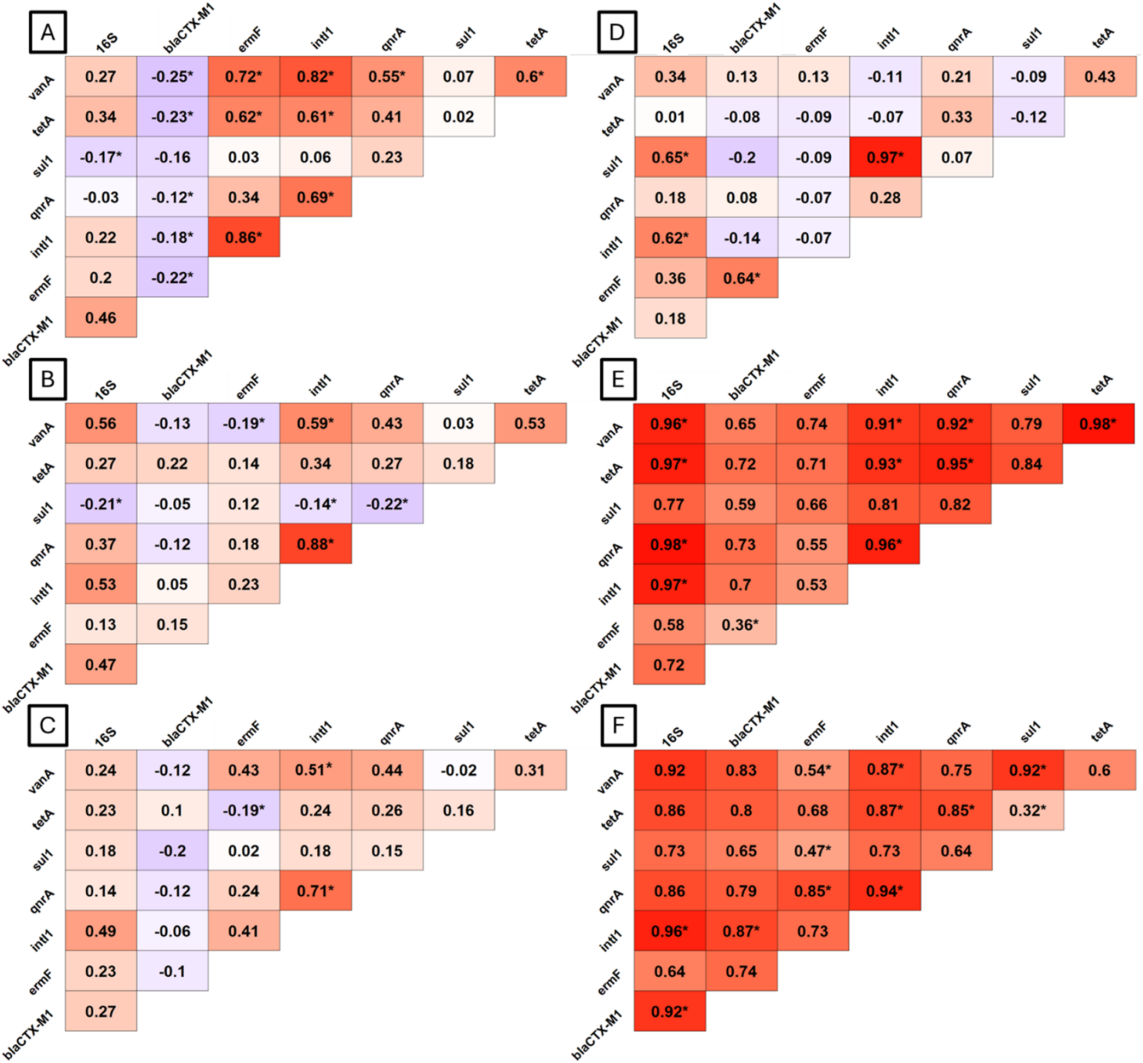
Spearman correlation correlograms of co-occurrence of targeted genes at the dairy farm (A) upwind, (B) 5 m downwind, and (C) combined downwind sites at 65 and 115 m, and at the swine farm at (D) road, (E) exhaust fan 1, and (F) exhaust fan 2 sites. Size-segregated gene concentrations were summed for each independent sampling day, with each correlogram representing 15–20 samples collected over the course of a year.

At the swine farm, strong correlations among ARGs at the exhaust fan sites were striking (Figure 3). At exhaust fan 1, *tetA*, *vanA*, *qnrA*, and *intI1* were all significantly correlated with each other (Figure 3E). Additionally, *bla*_*CTX‑M1*_ and *ermF* were correlated with each other, indicating a possible linkage in their dissemination pathways. At exhaust fan 2, *intI1*, *tetA*, and *qnrA* were significantly correlated with each other, while *vanA* was correlated with just *intI1* (Figure 3F). Additionally, *sul1, ermF*, and *vanA* were significantly correlated with each other, indicating a potential coselection mechanism where the presence of one ARG promotes the persistence of the other. Correlations among genes were fewer and weaker at the road location, where *intI1* and *sul1* were strongly and significantly correlated with each other, as were *bla*_*CTX‑M1*_ and *ermF* (Figure 3D).

Across the two farms, correlations with bacterial 16S rRNA genes were consistent with elevated bacteria in the air coinciding with elevated ARGs, significantly so at the swine farm for many genes close to the exhaust fans (Figure 3).

### ARG Particle Size Distributions

The size distributions of particles carrying the targeted genes are depicted in Figure 4, with concentrations in each size range normalized to the total concentration in each sample to facilitate comparison between locations and sampling days. Target gene concentrations from the second and third stages, and the fourth and fifth stages of the cascade impactor were combined to yield three categories of <1, 1–5, >5 μm, corresponding to fine, accumulation, and coarse mode particles, respectively. Target genes were associated with particles of all sizes, with high variability between samples. Upwind of the special-needs barn, many of the genes appeared to be depleted in coarse particles (Figure 4A). The fractions of *bla*_*CTX‑M1*_ and *ermF* in accumulation mode particles were significantly higher than in coarse particles, and the fractions of *bla*_*CTX‑M1*_ and *sul1* in fine particles were significantly higher than in coarse particles. 16S rRNA genes were significantly higher in the accumulation mode and fine particles than in the coarse particles at both the upwind and downwind sites. Target genes were noticeably enriched in coarse particles at the 5 m site compared to the others (Figure 4B). At this location just downwind of the special-needs barn, coarse particles accounted for ∼0.60 of the total concentration for all genes except for *tetA.* The fraction of genes in accumulation mode particles was significantly higher than in fine particles for *bla*_*CTX‑M1*_ and *intI1* at the 5 m site, centering around 0.30 of the total concentration. The fraction of genes in accumulation mode particles was significantly higher than in coarse particles downwind of the special-needs barn for *bla*_*CTX‑M1*_, *qnrA*, *tetA*, and *vanA* (Figure 4C). The fraction of genes in fine particles was also significantly higher than in coarse particles for *bla*_*CTX‑M1*_ at the downwind sites.

**Figure 4:**
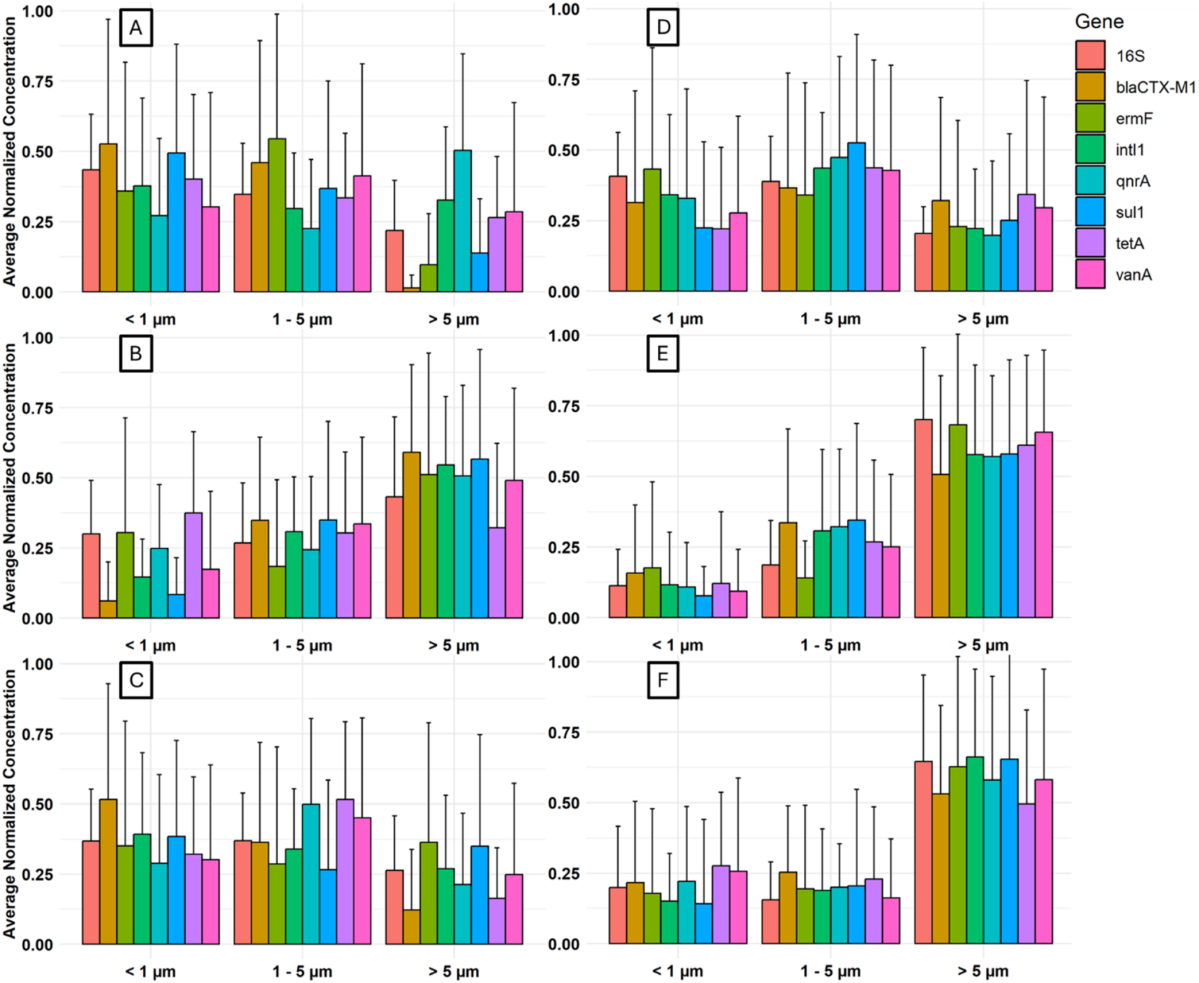
Normalized aerosol size distributions of targeted genes at the dairy farm (A) upwind, (B) 5 m downwind, and (C) combined downwind sites at 65 and 115 m, and at the swine farm at (D) road, (E) exhaust fan 1, and (F) exhaust fan 2 sites. Each plot represents 15–20 samples collected over the course of a year.

At exhaust fan 1, the fraction of targeted genes in coarse particles was significantly higher than in fine particles for all genes, and the fractions of all genes except for *bla*_*CTX‑M1*_ and *intI1* were significantly higher in coarse particles compared to accumulation mode particles (Figure 4E). The average fraction in coarse particles ranged from 0.50 for *bla*_*CTX‑M1*_ to 0.70 for *ermF*. At exhaust fan 2, all genes except *tetA* were significantly higher in coarse particles than in accumulation mode or fine particles (Figure 4F). The gene fraction in coarse particles ranged from 0.50 for *tetA* to 0.62 for *intI1*. At the road site, the targeted genes were more evenly distributed across the different size fractions (Figure 4D). Size distributions at the road site are described in more detail in the SI (Text S2).

### Emission Rates of ARGs

Estimation of emission rates at the dairy farm focused on three genes for which this farm appeared to be a source based on results described above: the 16S rRNA gene, *bla*_*CTX‑M1*_, and *intI1*. Emission rates were quantifiable on 10 to 12 out of 18 days, depending on the gene target, while other days were excluded due to missing data from a sampler malfunction, or low or undetectable concentrations. For *bla*_*CTX‑M1*_, emission rates ranged between 10^3^ and 10^4^ gc s^–1^. For *intI1*, emission rates ranged between 10^3^ to 10^5^ gc s^–1^. For 16S rRNA genes, emission rates ranged between 10^5^ and 10^7^ gc s^–1^. Among the other genes analyzed, only *vanA* had quantifiable emission rates on at least one-third of the days. An assessment of uncertainty in the estimated emission rates at the dairy farm is presented in the SI (Text S3).

For the swine farm, emission rates were directly calculated from the fan flow rates (4.5 m^3^ s^–1^ for both exhaust fans), exhaust fan run times, and the background-subtracted concentrations on 14 days when exhaust fan 1 was running and 13 days when exhaust fan 2 was running. For *bla*_*CTX‑M1*_ and *sul1*, emission rates ranged between 10^3^ and 10^5^ gc s^–1^. For *intI1*, emission rates ranged between 10^3^ and 10^6^ gc s^–1^. However, on 1 day, emissions of these three genes were negligible (i.e., not above background). For bacterial 16S rRNA genes, emission rates ranged between 10^6^ and 10^9^ gc s^–1^. *vanA*, *qnrA*, and *intI1* had peak emission rates on the order of 10^6^ gc s^–1^, which occurred on the same sampling day. These genes were also noted to be significantly correlated with each other. At exhaust fan 2, *bla*_*CTX‑M1*_, emission rates ranged between negligible and 10^5^ gc s^–1^. For *intI1*, *ermF*, *qnrA*, *sul1*, and *tetA*, emission rates ranged between negligible and 10^6^ gc s^–1^. For 16S rRNA, emission rates ranged between 10^5^ and 10^8^ gc s^–1^. *vanA* emission rates ranged between negligible and 10^6^ gc s^–1^. An exposure assessment for the target genes can be found in the SI (Figure S6 and Text S4).

## Discussion

This study employed air samplers at two distinct farm types, swine and dairy, to measure ARG concentrations in size-segregated aerosols as a function of location on the farm and other contributing factors. qPCR analysis provided precise estimates of a cross section of six ARGs and the class 1 integron, revealing clear spatial patterns and correlations indicating that animal facilities are a potential source of ARG emissions to the atmosphere through aerosolization of soil, litter, and dust. Examining trends across spring, summer, winter, and fall sampling events provided further insight into the range and variance in gene concentrations encountered.

The cross-section of genes examined here included ARGs that encode resistance to antibiotics deemed by the World Health Organization to be critically important (macrolide) to highest priority critically important (quinolone, extended-spectrum β lactams) in human and veterinary medicine, antibiotics only approved for use in humans (vancomycin), as well as antibiotics more routinely used in both humans and animals (tetracyclines). Compared to other environmental surveys of *bla*_*CTX‑M1*_ *, qnrA*, *vanA*, and *ermF* in aerosols, levels in air in this study were similarly in the range of 10^0^ to 10^6^ gc m^–3^ as reported in a variety of animal farm environments, affected by age, stock, breeding area, and type of animal.^16,35–38^ ARGs encoding resistance to macrolides, tetracyclines, aminoglycosides, and β-lactams had the highest concentrations observed in these studies, dependent on historical antibiotic usage at the farm, farm type, environmental conditions, and sampling location, all of which have been shown to contribute to variability in observed concentrations in many ARG studies.^5^ It is concerning to find all of these genes encoding resistance to clinically important antibiotics in the air around farms.

ARG concentrations were highest at locations nearest to the animal housing facilities, while lower concentrations were consistently found at sampling locations further upwind and downwind from the facilities (Figure 1). Downwind spatial trends observed for 16S rRNA genes, *bla*_*CTX‑M1*_, and *intI1* at the dairy farm align with the understanding that animal housing can be a major source of ARGs and bacterial emissions due to high bacterial loads from cows, fecal matter, and human activity, and antibiotic use.^39,40^ The relative abundance of *bla*_*CTX‑M1*_ maintained the same downwind spatial trend (Figure S3). Similar spatial trends have been observed in other studies, where ARG hotspots have been identified near sources such as livestock farms.^41^ The present study adds to this understanding by examining two distinct farm types in the same region utilizing conditional sampling to enhance spatial analysis, considering a range of key ARG types and contributing factors.

Higher concentrations of *intI1*, *ermF*, *qnrA*, and *tetA* during the summer at the dairy farm were particularly striking (Figure 1), while in terms of relative abundance, only *intI1* maintained this same trend (Figure S3). Bacterial numbers, as indicated by 16S rRNA gene levels, were also higher during the summer; this finding is consistent with warmer temperatures enhancing bacterial growth. These relationships are indicative of the influence of the farms as one of many potential sources that contribute to local bacterial loading in the air, with elevated levels of ARGs. Studies have shown that ARG concentrations in aquatic environments also peak during warmer months, implicating the wastewater storage tanks at the dairy farm as a possible reservoir for ARGs.^42^ A clear temporal pattern in airborne ARG concentrations was not observed at the swine farm; rather, with the sampling targeting exhaust fans directly exiting the barns, the number of animals and fan run time were the main drivers. Further, exhaust fan 1 served the facility that housed farrowing, nursing, and some finishing pigs, which receive more antibiotics than the pigs in the grower stage housed in the facility served by exhaust fan 2, and also consistently yielded air samples with higher concentrations of ARGs. Studies have shown that concentrations of ARGs are positively correlated with animal age, pen design, and stocking density, indicating that antibiotic usage is likely only one contributing factor to observed ARG concentrations in this study, among a complex array of factors and sources.^13,38^ Relative abundances were higher at the road site than at the two exhaust fans for all gene targets except *ermF* (Figure S5). The fact that longer fan run times enhanced emissions of ARGs to the atmosphere from both barns suggests that they provided greater opportunity for suspended particles to be exhausted outdoors rather than settle indoors. One straightforward way to control such emissions would be to place a filter or other particle control device on the exhaust fan.

Correlation analysis provided further insight into potential sources of airborne ARGs around the farms. Downwind *bla*_*CTX‑M1*_ concentrations strongly correlated with *bla*_*CTX‑M1*_ measured in the manure within the dairy’s special-needs barn where β-lactam antibiotics (ceftiofur) were regularly used, and *ermF* was strongly correlated as well, even though macrolides were not used during the study (Figure 2). At the swine farm, the strongest correlation among targeted airborne ARGs was with *bla*_*CTX‑M1*_ measured in the fan sill dust, but this was not the case for other genes. This could relate to the farm’s exclusive use of β-lactam antibiotics (penicillin and ceftiofur) during the study. Generational studies of antibiotic use in swine farms have noted selective enrichment of ARGs associated with tetracyclines, macrolides, and β-lactams (specifically penicillin) in manure.^43–45^ Airborne and soil concentrations were significantly correlated for *intI1*, *bla*_*CTX‑M1*_, and *tetA*, indicating that soil may act as a reservoir for these genes and contribute to their aerosolization, or act as a sink for the larger particles emitted from the exhaust fan. Soil, particularly in agricultural settings, can harbor high levels of ARGs and MGEs due to contamination from animal waste, human activities, and horizontal gene transfer.^42,46^ The proximity of the swine farm to other potential sources, such as the campus and nearby town, may contribute to the presence of *intI1* and *sul1*, especially at the road site, as these genes are well established as indicators of anthropogenic sources of antibiotic resistance, and generally dominant in populated areas.^5,47,48^

It is also noteworthy that sampling took place during the spring and summer of 2023, when wildfires in Canada affected PM_2.5_ levels in the sampling area.^49^ Wildfires have been shown to increase ARG concentrations due to the elevated levels of particulate matter and other pollutants they introduce into the atmosphere, and through increased horizontal gene transfer induced by stressful conditions for the bacteria.^50^ This additional environmental stressor may have contributed to some of the variations in targeted gene levels during the sampling period. Three sampling days were impacted by the wildfire smoke, two in the spring and one in the summer, with PM_2.5_ concentrations near 40 μg m^–3^, very high for a region where levels rarely exceed the National Ambient Air Quality Standard of 35 μg m^–3^ (24-h average). Other studies have noted an association of air pollution with elevated ARGs.^51^ Soil and wastewater are also typical reservoirs for ARGs, and the correlation of ARGs in these matrices with airborne ones at different locations on the dairy farm suggests a complex interplay between different environmental compartments.^52,53^

The genes targeted here correlated more strongly with each other at sampling locations nearest to the barns, as was especially apparent when sampling near the exhaust fans from the swine barns (Figure 3). This is consistent with the animals and their manure being the primary source of airborne ARGs. Interestingly, *intI1* and *sul*1 stood out as being most strongly correlated 50 m away from the swine facility and also were the only genes significantly positively correlated with wind speed. This suggests other contributing sources of *intI1* and *sul*1 and is consistent with these genes being prevalent indicators of anthropogenic sources of AMR,^12,24,25^ correspondingly providing a useful measure of background sources on the farms. Other studies have found *intI1* and *sul1* to be abundant in the atmosphere across a variety of environments.^54^ Class 1 integrons are also a useful indicator of mobile, multi antibiotic resistance.^25^ Elevated levels of *intI1* indicate active gene transfer processes, potentially exacerbated by environmental stressors like temperature and antibiotic use.^41^

Some of these ARGs might occur on the same genetic element and thus can be coselected even if unrelated antibiotics are used. The presence of *ermF* and *tetA* despite their lack of recent use could be due to historical use of macrolides and tetracyclines, other background sources on the farm, or influence from surrounding farms and facilities.^55^ The correlation between *bla*_*CTX‑M1*_ and *ermF* suggests that these genes might have similar sources and dissemination pathways; they could possibly be present on some of the same genetic elements and thus *ermF* could be coselected by the dominant use of β-lactams at the farm during the study period, while *bla*_*CTX‑M1*_ could be impacted by the many potential sources of *ermF* and *tetA*. A recent study examining the correlation of multiple ARGs in swine manure across multiple US and Chinese farms suggests that clusters, defined as groups of resistance and mobile genetic element alleles that co-occur, are particularly enriched in swine feces.^43^ These clusters contain a multitude of resistance genes that persist independently of the specific antibiotics used on the farm. Despite this, the enrichment of these clusters still appears to be linked to the overall antibiotic and chemical usage patterns at the farm. Overall, the findings of this study are consistent with the understanding that there are many potential sources and drivers of observed airborne ARGs on farms outside of current antibiotic usage patterns.

Size distribution analysis revealed a dominance of coarse particles near the source for both farms (Figure 4), which aligns with the understanding that larger particles are more likely to be detected near emission sources because they settle to the ground before being able to travel farther.^38,56,57^ Detection of ARGs encoding resistance to clinically relevant antibiotics in accumulation mode and fine particles emitted from both farms is concerning because such particles may be transported tens of kilometers or more in the atmosphere.^58^ There were almost no instances at the upwind location of detection of *bla*_*CTX‑M1*_ in coarse particles, and very few for *ermF* and *sul1*. Presumably, the upwind samples represented background levels that were transported longer distances, as there was no obvious source near the sampling site, so coarse particles would have had more of an opportunity for removal by gravitational settling by the time they reached this site. The significantly higher concentrations of 16S rRNA genes in accumulation mode and fine particles confirms that bacterial DNA, including ARGs, in these samples is associated with smaller particles that can remain airborne and be transported over longer distances.

The higher emission rates for *bla*_*CTX‑M1*_ and *intI1* suggest that these genes were more effectively dispersed from the dairy farm, possibly due to their association with specific bacterial hosts, environmental conditions that favor their aerosolization, presence in all particle sizes, and the use of β-lactams on the farm.^59^ Due to the small amount of data available, fluctuations in environmental conditions, approximation of the farm as a point source, background contributions from other sources,^42^ and other limitations, a large amount of uncertainty is associated with the estimated emission rates. Nonetheless, as a model for future studies, we have demonstrated approaches to quantify emissions from agricultural sources. Such estimates, even if their accuracy is limited to an order of magnitude, are critical for understanding the relative contributions of different sources to atmospheric loading of bacteria and ARGs. The estimated emission rates for both farms fall within the ranges reported through modeling and laboratory testing in prior studies for ARGs and bacterial 16S rRNA genes near farms.^15,60^

The emission rates of *bla*_*CTX‑M1*_ at the open-air dairy farm and the mechanically ventilated swine farm may have been influenced by several factors. First, the number of animals housed at each facility likely played a significant role; a higher number of cows at the dairy farm compared to pigs at the swine farm may have led to a higher bacterial load and greater opportunities for the spread of *bla*_*CTX‑M1*_. The layout and size of the facilities may also have influenced emissions, as the more expansive layout of the dairy farm provided more opportunities for aerosolization of *bla*_*CTX‑M1*_ from wastewater and soil. Antibiotic usage could also be a key driver. Variability in the use of β-lactam antibiotics, which can potentially select for *bla*_*CTX‑M1*_, may have affected emission rates. Greater use for a longer duration could produce higher emission rates. Presence of heavy metals, soil conditions, and weather conditions, could also affect the selective pressure for dissemination of certain ARGs to the atmosphere, but these factors were not measured in this study. Additionally, cleaning and hygiene protocols and other human activities at each facility may have impacted transmission sources of multidrug resistant bacteria and mobile genetic elements.^15,60^ Differences in sampling methods, time, and environmental conditions during sampling may have introduced variability in measured emission rates, potentially masking expected differences between natural and mechanical ventilation systems.

This study contributes toward the database of concentrations of ARGs in air and adds critical information about the size of particles carrying ARGs. The study also demonstrates approaches for estimating emissions of ARGs to the atmosphere from agricultural sources. Observed spatial patterns provide evidence for these farms as sources of emissions to the atmosphere of certain ARGs. Due to limitations of the study, temporal variability and estimates of emissions carry greater uncertainty. The sampling frequency and duration, limited to 4 or 5 days per season, does not capture the full variability of ARG emissions throughout the year, and the 24 h sampling period misses short-term fluctuations. Wind and weather conditions during the study also might not have captured the full range of typical conditions. Variability in animal populations, particularly the fluctuating number and ages of pigs, could affect ARG emissions. The fixed number of samplers limits spatial resolution, and the reliance on conditional sampling based on wind direction and speed could introduce biases. The use of qPCR for ARG detection does not distinguish between viable and nonviable bacteria. The inverse dispersion modeling approach for estimating emission rates does not capture hourly variations and does not account for particle deposition, although reliance on observations at 65 and 115 m from the source focuses the estimates on accumulation mode and fine particles that can be transported longer distances.

The findings of this study underscore the need for targeted strategies to mitigate ARG spread in agricultural settings, emphasizing the importance of monitoring various particle sizes and understanding the environmental dynamics that influence ARG dissemination. Future studies may conduct chemical analyses on water and soil samples to identify additional environmental correlations with airborne bacteria, and further metagenomic analysis could uncover additional microbial ecological factors at play. A more focused study of the effects of antibiotic and antimicrobial use on airborne ARG dissemination would also be of value. Such information could contribute toward the development of a process-based model to predict emissions of ARGs in a variety of agricultural settings.

## Supplementary Material



## Notes

Description of the Gaussian dispersion model; qPCR protocols and primers for each targeted gene; overhead view of the farms with sampling locations; photographs of the custom conditional air sampler package; average and total gene concentrations by both season and location at the dairy farm; average gene concentrations for all sites at the swine farm, sorted by fan run times; description of size distributions at the road site evaluation of uncertainty in emission rates derived from Gaussian dispersion model (PDF)

## References

[1] World Health Organization . WHO Report on Surveillance of Antibiotic Consumption 2018 https://www.who.int/publications/i/item/who-report-on-surveillance-of-antibiotic-consumption.

[2] World Health Organization . Global Antimicrobial Resistance and Use Surveillance System (GLASS) Report 2022 www.who.int/publications/i/item/9789240062702.

[3] Chen P., Guo X., Li F. (2022). Antibiotic
Resistance Genes in Bioaerosols: Emerging,
Non-Ignorable and Pernicious Pollutants. J.
Cleaner Prod..

[4] Lee G., Yoo K. (2022). A Review of the Emergence of Antibiotic Resistance
in Bioaerosols
and Its Monitoring Methods. Rev. Environ. Sci.
Bio/Technol..

[5] Kormos D., Lin K., Pruden A., Marr L. C. (2022). Critical
Review of Antibiotic Resistance
Genes in the Atmosphere. Environ. Sci. Process.
Impacts.

[6] Mazar Y., Cytryn E., Erel Y., Rudich Y. (2016). Effect of Dust Storms
on the Atmospheric Microbiome in the Eastern Mediterranean. Environ. Sci. Technol..

[7] Gat D., Mazar Y., Cytryn E., Rudich Y. (2017). Origin-Dependent Variations
in the Atmospheric Microbiome Community in Eastern Mediterranean Dust
Storms. Environ. Sci. Technol..

[8] Pupo M., Pisano A., Lappano R., Santolla M. F., Francesco E. M., Abonante S., Rosano C., Maggiolini M. (2012). Antibiotics,
Bacteria, and Antibiotic Resistance Genes: Aerial Transport from Cattle
Feed Yards via Particulate Matter. Environ.
Health Perspect..

[9] Thames C. H., Pruden A., James R. E., Ray P. P., Knowlton K. F. (2012). Excretion
of Antibiotic Resistance Genes by Dairy Calves Fed Milk Replacers
with Varying Doses of Antibiotics. Front. Microbiol..

[10] Chambers L., Yang Y., Littier H., Ray P., Zhang T., Pruden A., Strickland M., Knowlton K. (2015). Metagenomic Analysis
of Antibiotic Resistance Genes in Dairy Cow Feces Following Therapeutic
Administration of Third Generation Cephalosporin. PLoS One.

[11] Vikesland P. J., Pruden A., Alvarez P. J. J., Aga D., Bürgmann H., Li X. D., Manaia C. M., Nambi I., Wigginton K., Zhang T., Zhu Y. G. (2017). Toward a Comprehensive
Strategy to
Mitigate Dissemination of Environmental Sources of Antibiotic Resistance. Environ. Sci. Technol..

[12] Keenum I., Liguori K., Calarco J., Davis B. C., Milligan E., Harwood V. J., Pruden A. (2022). A Framework
for Standardized QPCR-Targets
and Protocols for Quantifying Antibiotic Resistance in Surface Water,
Recycled Water and Wastewater. Crit. Rev. Environ.
Sci. Technol..

[13] Pholwat S., Pongpan T., Chinli R., McQuade E. T. R., Thaipisuttikul I., Ratanakorn P., Liu J., Taniuchi M., Houpt E. R., Foongladda S. (2020). Antimicrobial
Resistance in Swine Fecal Specimens Across
Different Farm Management Systems. Front. Microbiol..

[14] Yan H., Zhang L., Guo Z., Zhang H., Liu J. (2019). Production
Phase Affects the Bioaerosol Microbial Composition and Functional
Potential in Swine Confinement Buildings. Animals.

[15] Zhang J., Lu T., Chai Y., Sui Q., Shen P., Wei Y. (2019). Which Animal
Type Contributes the Most to the Emission of Antibiotic Resistance
Genes in Large-Scale Swine Farms in China?. Sci. Total Environ..

[16] Sancheza H. M., Echeverria C., Thulsiraj V., Zimmer-Faust A., Flores A., Laitz M., Healy G., Mahendra S., Paulson S. E., Zhu Y., Jay J. A. (2016). Antibiotic Resistance
in Airborne Bacteria Near Conventional and Organic Beef Cattle Farms
in California, USA. Water, Air, Soil Pollut..

[17] Pilote J., Létourneau V., Girard M., Duchaine C. (2019). Quantification of Airborne
Dust, Endotoxins, Human Pathogens and Antibiotic and Metal Resistance
Genes in Eastern Canadian Swine Confinement Buildings. Aerobiologia.

[18] Heckendorn, S. Soil Sample Information Sheet for Home Lawns, Gardens, Fruits, and Ornamentals. In Communications and Marketing CoAaLS; Virginia Tech, 2021 https://www.pubs.ext.vt.edu/452/452-125/452-125.html.

[19] Li A. D., Metch J. W., Wang Y., Garner E., Zhang A. N., Riquelme M. V., Vikesland P. J., Pruden A., Zhang T. (2018). Effects of
Sample Preservation and DNA Extraction on Enumeration of Antibiotic
Resistance Genes in Wastewater. FEMS Microbiol
Ecol.

[20] Colomer-Lluch M., Jofre J., Muniesa M. (2014). Quinolone Resistance Genes (QnrA
and QnrS) in Bacteriophage Particles from Wastewater Samples and the
Effect of Inducing Agents on Packaged Antibiotic Resistance Genes. J. Antimicrob. Chemother..

[21] Chen J., Yu Z., Michel F. C., Wittum T., Morrison M. (2007). Development and Application
of Real-Time PCR Assays for Quantification of Erm Genes Conferring
Resistance to Macrolides-Lincosamides-Streptogramin B in Livestock
Manure and Manure Management Systems. Appl.
Environ. Microbiol..

[22] Goldstein C., Lee M. D., Sanchez S., Hudson C., Phillips B., Register B., Grady M., Liebert C., Summers A. O., White D. G., Maurer J. J. (2001). Incidence
of Class 1 and 2 Integrases
in Clinical and Commensal Bacteria from Livestock, Companion Animals,
and Exotics. Antimicrob. Agents Chemother..

[23] Suzuki M. T., Taylor L. T., DeLong E. F. (2000). Quantitative Analysis of Small-Subunit
RRNA Genes in Mixed Microbial Populations via 5′-Nuclease Assays. Appl. Environ. Microbiol..

[24] Davis B. C., Riquelme M. V., Ramirez-Toro G., Bandaragoda C., Garner E., Rhoads W. J., Vikesland P., Pruden A. (2020). Demonstrating an Integrated Antibiotic Resistance Gene
Surveillance Approach in Puerto Rican Watersheds Post-Hurricane Maria. Environ. Sci. Technol..

[25] Gillings M. R., Gaze W. H., Pruden A., Smalla K., Tiedje J. M., Zhu Y. G. (2015). Using the Class
1 Integron-Integrase Gene as a Proxy
for Anthropogenic Pollution. ISME J..

[26] Moodley A., Guardabassi L. (2009). Transmission of IncN Plasmids Carrying BlaCTX-M-1 between
Commensal in Pigs
and Farm Workers. Antimicrob. Agents Chemother..

[27] Kanwar N., Scott H. M., Norby B., Loneragan G. H., Vinasco J., McGowan M., Cottell J. L., Chengappa M. M., Bai J., Boerlin P. (2013). Effects of Ceftiofur and Chlortetracycline Treatment
Strategies on Antimicrobial Susceptibility and on Tet­(A), Tet­(B),
and BlaCMY-2 Resistance Genes among Isolated from the Feces of Feedlot Cattle. PLoS One.

[28] Kanwar N., Scott H. M., Norby B., Loneragan G. H., Vinasco J., Cottell J. L., Chalmers G., Chengappa M. M., Bai J., Boerlin P. (2014). Impact of Treatment Strategies on Cephalosporin and
Tetracycline Resistance Gene Quantities in the Bovine Fecal Metagenome. Sci. Rep.

[29] Weinroth M. D., Scott H. M., Norby B., Loneragan G. H., Noyes N. R., Rovira P., Doster E., Yang X., Woerner D. R., Morley P. S., Belk K. E. (2018). Effects of Ceftiofur
and Chlortetracycline on the Resistomes of Feedlot Cattle. Appl. Environ. Microbiol..

[30] Taha M. P. M., Drew G. H., Longhurst P. J., Smith R., Pollard S. J. T. (2006). Bioaerosol
Releases from Compost Facilities: Evaluating Passive and Active Source
Terms at a Green Waste Facility for Improved Risk Assessments. Atmos. Environ..

[31] Dowd S. E., Gerba C. P., Pepper I. L., Pillai S. D. (2000). Bioaerosol Transport
Modeling and Risk Assessment in Relation to Biosolid Placement. J. Environ. Qual..

[32] Pfender W., Graw R., Bradley W., Carney M., Maxwell L. (2006). Use of a Complex
Air Pollution Model to Estimate Dispersal and Deposition of Grass
Stem Rust Urediniospores at Landscape Scale. Agric. For. Meteorol..

[33] Jahne M. A., Rogers S. W., Holsen T. M., Grimberg S. J. (2015). Quantitative Microbial
Risk Assessment of Bioaerosols from a Manure Application Site. Aerobiologia.

[34] Cichowicz R., Wielgosiński G., Fetter W. (2020). Effect of Wind Speed
on the Level
of Particulate Matter PM10 Concentration in Atmospheric Air during
Winter Season in Vicinity of Large Combustion Plant. J. Atmos Chem..

[35] Song L., Wang C., Jiang G., Ma J., Li Y., Chen H., Guo J. (2021). Bioaerosol Is an Important
Transmission
Route of Antibiotic Resistance Genes in Pig Farms. Environ. Int..

[36] Xin H., Qiu T., Guo Y., Gao H., Zhang L., Gao M. (2023). Aerosolization
Behavior of Antimicrobial Resistance in Animal Farms: A Field Study
from Feces to Fine Particulate Matter. Front.
Microbiol..

[37] Xin H., Gao M., Wang X., Qiu T., Guo Y., Zhang L. (2022). Animal Farms
Are Hot Spots for Airborne Antimicrobial Resistance. Sci. Total Environ..

[38] Bai Y., Sun X., Guo Y., Qiu T., Xin H., Yu A., Wang X., Gao M. (2023). Particle-Size
Stratification of Airborne
Antibiotic Resistant Genes, Mobile Genetic Elements, and Bacterial
Pathogens within Layer and Broiler Farms in Beijing, China. Environ. Sci. Pollut. Res..

[39] Smith S. D., Colgan P., Yang F., Rieke E. L., Soupir M. L., Moorman T. B., Allen H. K., Howe A. (2019). Investigating the Dispersal
of Antibiotic Resistance Associated Genes from Manure Application
to Soil and Drainage Waters in Simulated Agricultural Farmland Systems. PLoS One.

[40] Chee-Sanford J. C., Mackie R. I., Koike S., Krapac I. G., Lin Y., Yannarell A. C., Maxwell S., Aminov R. I. (2009). Fate and Transport
of Antibiotic Residues and Antibiotic Resistance Genes Following Land
Application of Manure Waste. J. Environ. Qual.

[41] Cheng G., Ning J., Ahmed S., Huang J., Ullah R., An B., Hao H., Dai M., Huang L., Wang X., Yuan Z. (2019). Selection and Dissemination of Antimicrobial Resistance in Agri-Food
Production. Antimicrob. Resist. and Infection
Control.

[42] McKinney C. W., Dungan R. S., Moore A., Leytem A. B. (2018). Occurrence and Abundance
of Antibiotic Resistance Genes in Agricultural Soil Receiving Dairy
Manure. FEMS Microbiol. Ecol..

[43] Johnson T. A., Stedtfeld R. D., Wang Q., Cole J. R., Hashsham S. A., Looft T., Zhu Y. G., Tiedje J. M. (2016). Clusters of Antibiotic
Resistance Genes Enriched Together Stay Together in Swine Agriculture. mBio.

[44] Looft T., Johnson T. A., Allen H. K., Bayles D. O., Alt D. P., Stedtfeld R. D., Sul W. J., Stedtfeld T. M., Chai B., Cole J. R., Hashsham S. A., Tiedje J. M., Stanton T. B. (2012). In-Feed Antibiotic Effects on the Swine Intestinal
Microbiome. Proc. Natl. Acad. Sci. U.S.A..

[45] Neher T. P., Soupir M. L., Andersen D. S., O’Neill M. L., Howe A. (2023). Comparison of Antibiotic Resistance Genes in Swine Manure Storage
Pits of Iowa, USA. Front. Antibiotics.

[46] Ondon B. S., Li S., Zhou Q., Li F. (2021). Sources of Antibiotic Resistant Bacteria
(ARB) and Antibiotic Resistance Genes (ARGs) in the Soil: A Review
of the Spreading Mechanism and Human Health Risks. Rev. Environ. Contam. Toxicol..

[47] Fogler K., Guron G. K. P., Wind L. L., Keenum I. M., Hession W. C., Krometis L. A., Strawn L. K., Pruden A., Ponder M. A. (2019). Microbiota
and Antibiotic Resistome of Lettuce Leaves and Radishes Grown in Soils
Receiving Manure-Based Amendments Derived From Antibiotic-Treated
Cows. Front Sustainable Food Syst..

[48] Hoa P. T. P., Nonaka L., Viet P. H., Suzuki S. (2008). Detection of the Sul1,
Sul2, and Sul3 Genes in Sulfonamide-Resistant Bacteria from Wastewater
and Shrimp Ponds of North Vietnam. Sci. Total
Environ..

[49] Shakoor A., Farooq T. H., Arif M. S., Shahzad S. M. (2023). Unprecedented Wildfires
in Canada and Transboundary Effects of Carbon Monoxide Pollution. Nat. Hazards.

[50] Li T., Cui L., Liu L., Chen Y., Liu H., Song X., Xu Z. (2023). Advances in
the Study of Global Forest Wildfires. J. Soils
Sediments.

[51] Pal C., Bengtsson-Palme J., Kristiansson E., Larsson D. G. J. (2016). The Structure
and Diversity of Human, Animal and Environmental Resistomes. Microbiome.

[52] Han B., Ma L., Yu Q., Yang J., Su W., Hilal M. G., Li X., Zhang S., Li H. (2022). The Source,
Fate and Prospect of
Antibiotic Resistance Genes in Soil: A Review. Front. Microbiol..

[53] Li H., Lin Y., Qin X., Song L., Fan F., Liu Y., Li S. (2024). An Updated Review on How Biochar May Possess Potential
in Soil ARGs
Control on Aspects of Source, Fate and Elimination. Biochar.

[54] Marshall B. M., Levy S. B. (2011). Food Animals and Antimicrobials:
Impacts on Human Health. Clin. Microbiol. Rev..

[55] Rothrock M. J., Min B. R., Castleberry L., Waldrip H., Parker D., Brauer D., Pitta D., Indugu N. (2021). Antibiotic Resistance,
Antimicrobial Residues, and Bacterial Community Diversity in Pasture-Raised
Poultry, Swine, and Beef Cattle Manures. J.
Anim Sci..

[56] Chen M., Qiu T., Sun Y., Song Y., Wang X., Gao M. (2019). Diversity
of Tetracycline- and Erythromycin-Resistant Bacteria in Aerosols and
Manures from Four Types of Animal Farms in China. Environ. Sci. Pollut. Res..

[57] Gao M., Jia R., Qiu T., Han M., Wang X. (2017). Size-Related Bacterial
Diversity and Tetracycline Resistance Gene Abundance in the Air of
Concentrated Poultry Feeding Operations. Environ.
Pollut..

[58] Falcon-Rodriguez C. I., Osornio-Vargas A. R., Sada-Ovalle I., Segura-Medina P. (2016). Aeroparticles,
Composition, and Lung Diseases. Front. Immunol..

[59] Salerno B., Cornaggia M., Sabatino R., Di Cesare A., Furlan M., Barco L., Orsini M., Cordioli B., Mantovani C., Bano L., Losasso C. (2022). Calves as Main Reservoir
of Antibiotic Resistance Genes in Dairy Farms. Front. Public Health.

[60] Baghdadi M., Brassard P., Godbout S., Létourneau V., Turgeon N., Rossi F., Lachance É., Veillette M., Gaucher M. -L., Duchaine C. (2023). Contribution of Manure-Spreading
Operations to Bioaerosols and Antibiotic Resistance Genes’
Emission. Microorganisms.

