# Supplementary material for: Quantifying Dissemination of Antibiotic Resistance Genes in Air from a Dairy Farm and Swine Farm
Source: ACS EST Air. 2025 Jul 18;2(8):1552–64. doi: 10.1021/acsestair.5c00055 (PMC12340760; doi:10.1021/acsestair.5c00055)
Supplement: Supplementary file 1 [file ea5c00055_si_001.pdf]

Supporting Information

Quantifying dissemination of antibiotic resistance genes in air from a dairy farm and swine farm

David A. Kormos[1], Gabriel Isaacman-VanWertz[1], Jactone A. Ogejo[2], Amy Pruden[1], Linsey C. Marr [1]

[1] Department of Civil and Environmental Engineering, Virginia Tech, Blacksburg, Virginia, USA 24061

[2] Department of Biological Systems Engineering, Virginia Tech, Blacksburg, Virginia, USA 24061

Text S1. Description of the Gaussian dispersion model.

For the dairy farm, we utilized the Gaussian dispersion model shown in Equation 1 to estimate emission rates:

$$C(x,y,z) = \frac{q}{2\pi u \sigma_y \sigma_z} \exp\left(-\frac{y^2}{2\sigma_y^2}\right)\left\{\exp\left(-\frac{(z-h)^2}{2\sigma_z^2}\right) + \exp\left(-\frac{(z+h)^2}{2\sigma_z^2}\right)\right\} \quad (1)$$

where C is the above-background concentration, x is distance in the downwind direction, y is distance in the cross-wind direction, z is height above ground, q is the emission rate, u is wind speed, h is the release height (assumed to be ground level for the open-air, special-needs barn), and σ_y and σ_z are the dispersion parameters in the y and z directions, respectively. We used the average wind speed from each sampler while it was sampling. We calculated the dispersion coefficients as a function of x using parameterizations of the Pasquill curves.[1] Given observations of C and the other parameters, one can solve Eq. 1 for q. Alternatively, because the relationship between C and

q is linear, one can calculate C for a unit emission rate and then infer q by multiplying by C_{obs}/C_{pred}, the ratio of the observed to unit-mass-predicted concentrations.[2] We use a version of the latter approach here, calculating the predicted concentrations for a unit emissions rate, and taking the optimum emission rate as the slope of the correlation between observed and predicted concentrations at both sites (65 m and 115 m).

Table S1. qPCR protocols and primers for each targeted gene.

Gene	Annealing/Extension	Primer Sequence (5′-3′)	Amplicon length (bp)
16S rRNA[3]	98°C for 2min (98°C for 5s, 55°C for 5s) x40 cycles	CGGTGAATACGTTCYCGG<br>GGWTACCTTGTTACGACTT	123
bla$_{CTX\text{-}M1}$[4]	98°C for 2min (98°C for 5s, 55°C for 30s, 72°C for 30s) x40 cycles	CGTCACGCTGTTGTTAGGAA<br>ACGGCTTTCTGCCTTAGGTT	780
ermF[5]	98°C for 2min (98°C for 5s, 56°C for 15s, 72°C for 15s) x40 cycles	CGACACAGCTTTGGTTGAAC<br>GGACCTACCTCATAGACAAG	309
intI1[6]	98°C for 2min (98°C for 5s, 58°C for 5s) x40 cycles	CCTCCCGCACGATGATC<br>TCCACGCATCGTCAGGC	280
qnrA[7]	98°C for 2min (98°C for 5s, 56°C for 15s, 72°C for 15s) x40 cycles	AGAGGATTTCTCACGCCAGG<br>TGCCAGGCACAGATCTTGAC	580
sul1[8]	98°C for 2min (98°C for 5s, 69.9°C for 5s) x40 cycles	CGCACCGGAAACATCGCTGCAC<br>TGAAGTTCCGCCGCAAGGCTCG	163
tetA[9]	98°C for 2min (98°C for 5s, 60°C for 5s) x40 cycles	GCTACATCCTGCTTGCCTTC<br>CATAGATCGCCGTGAAGAGG	210
vanA[10]	98°C for 2min (98°C for 5s, 60°C for 30s, 72°C for 30s) x40 cycles	GGGAAAACGACAATTGC<br>GTACAATGCGGCCGTTA	732

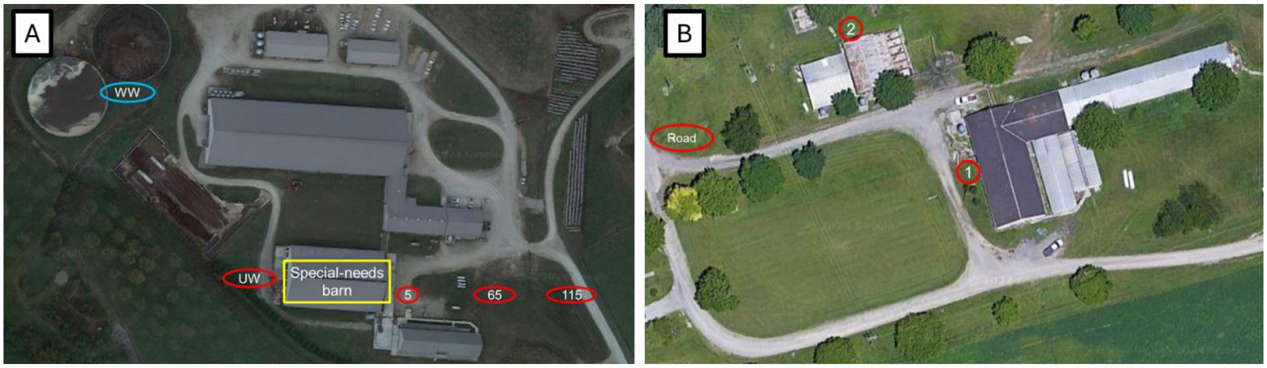

Figure S1. Overhead view of the (A) dairy farm with air sampling locations upwind (UW) and 5, 65, and 115 m downwind of the special-needs barn and the liquid manure storage tank, represented as wastewater (WW), and the (B) swine farm with sampling locations exhaust fan 1, exhaust fan 2, and the road site.

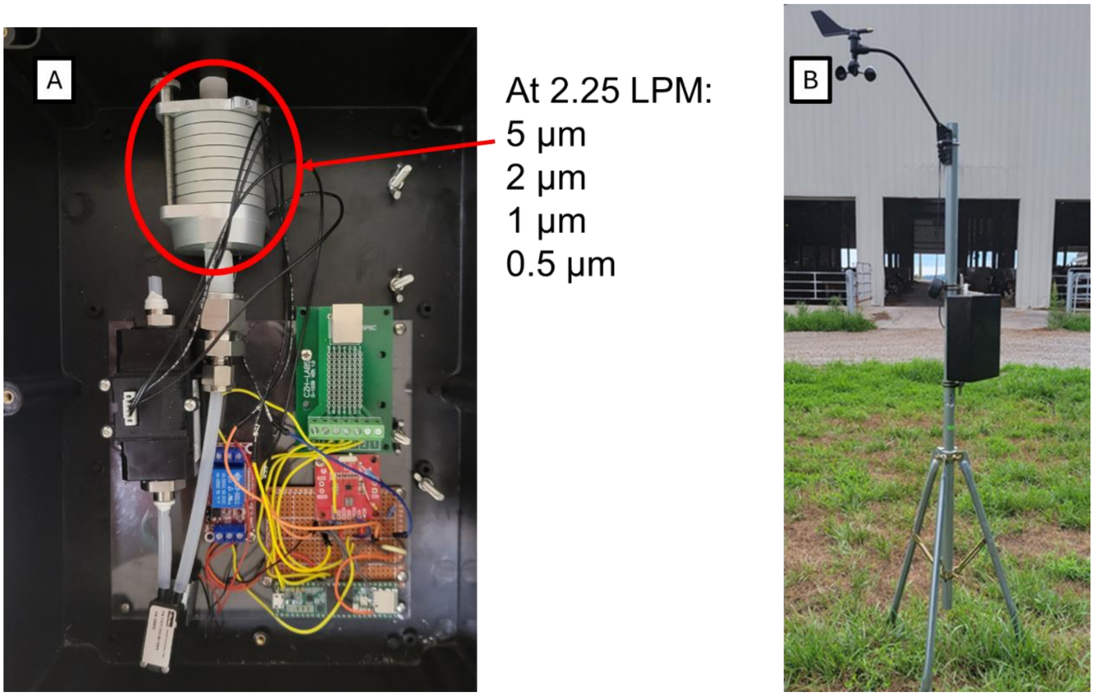

Figure S2. Custom conditional air sampler package (A) with the cascade impactor and size cuts for each stage highlighted and (B) in the field with the anemometer and vane attachment.

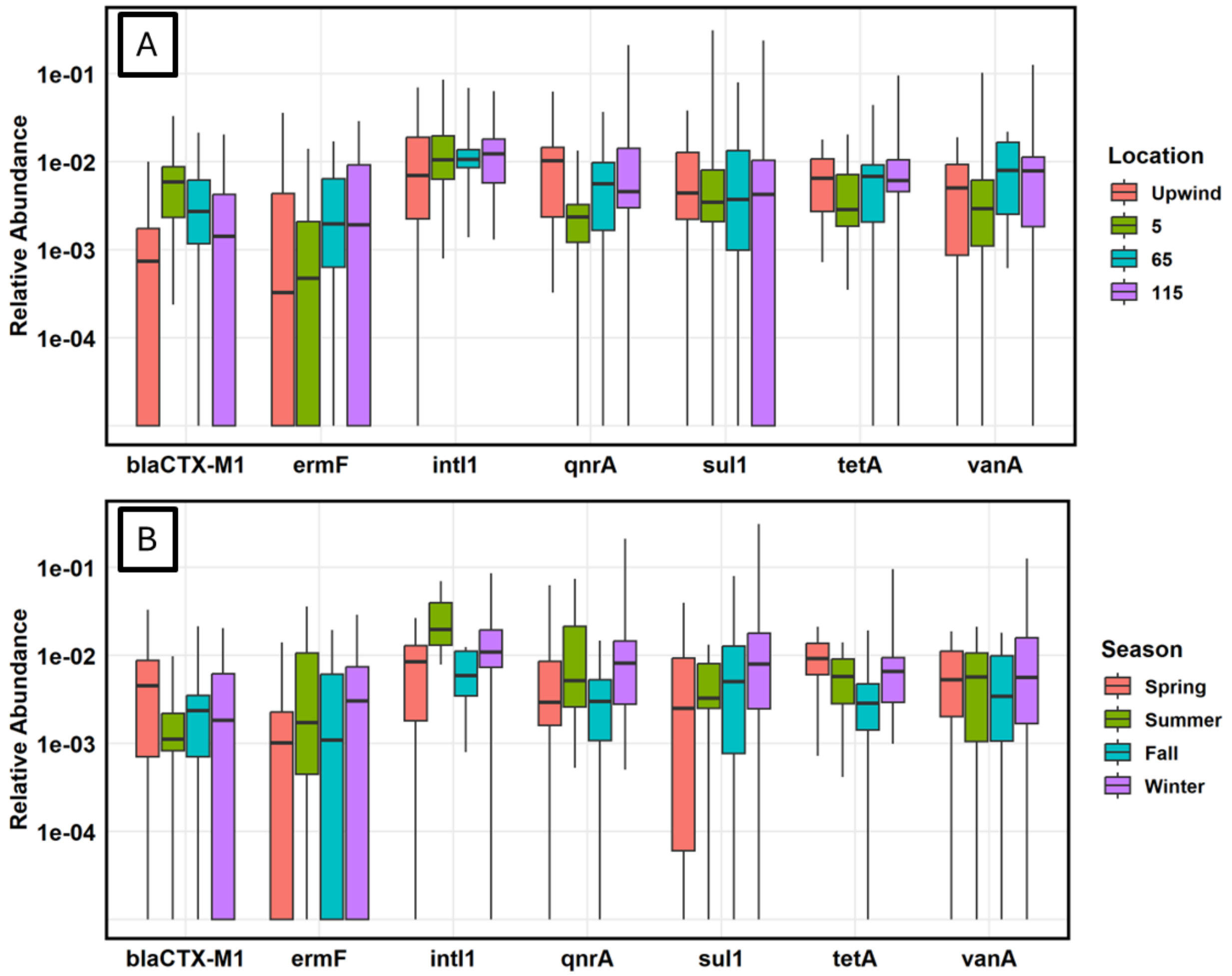

Figure S3. Relative abundance of each targeted gene (proportion of gene copies of the target to those of the 16S rRNA gene representing total bacteria) in air samples at the dairy farm, (A) by location (Upwind; 5, 65, and 115 m downwind of the special-needs barn) and (B) by the season of the sampling event.

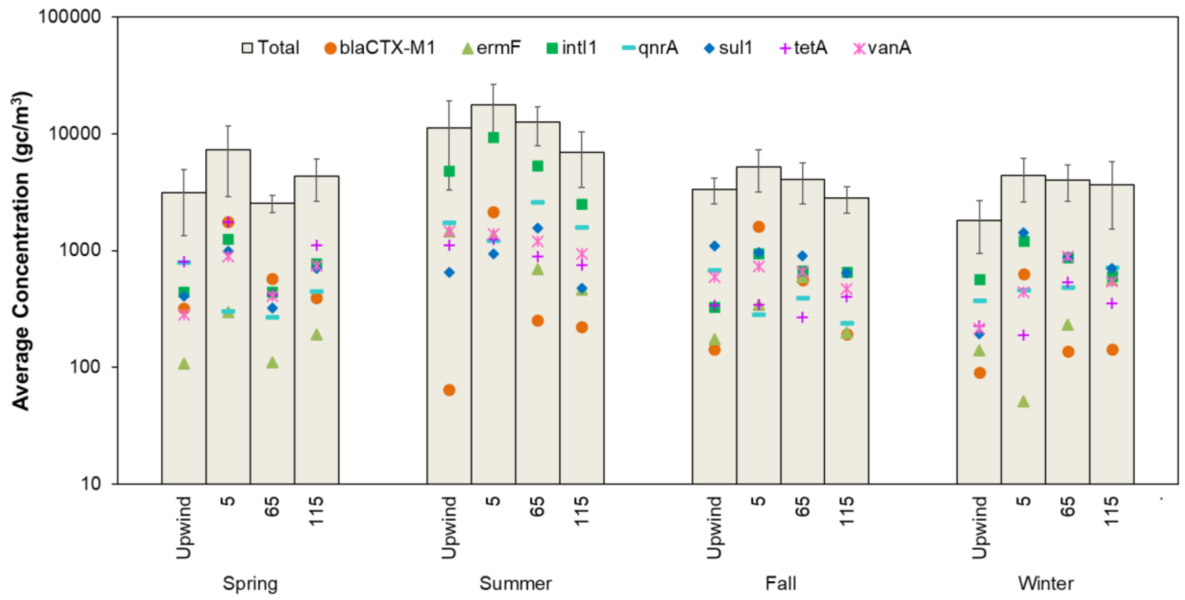

Figure S4. Average and total gene concentrations by both season and location (Upwind; 5, 65, and 115 m downwind of the special-needs barn) at the dairy farm.

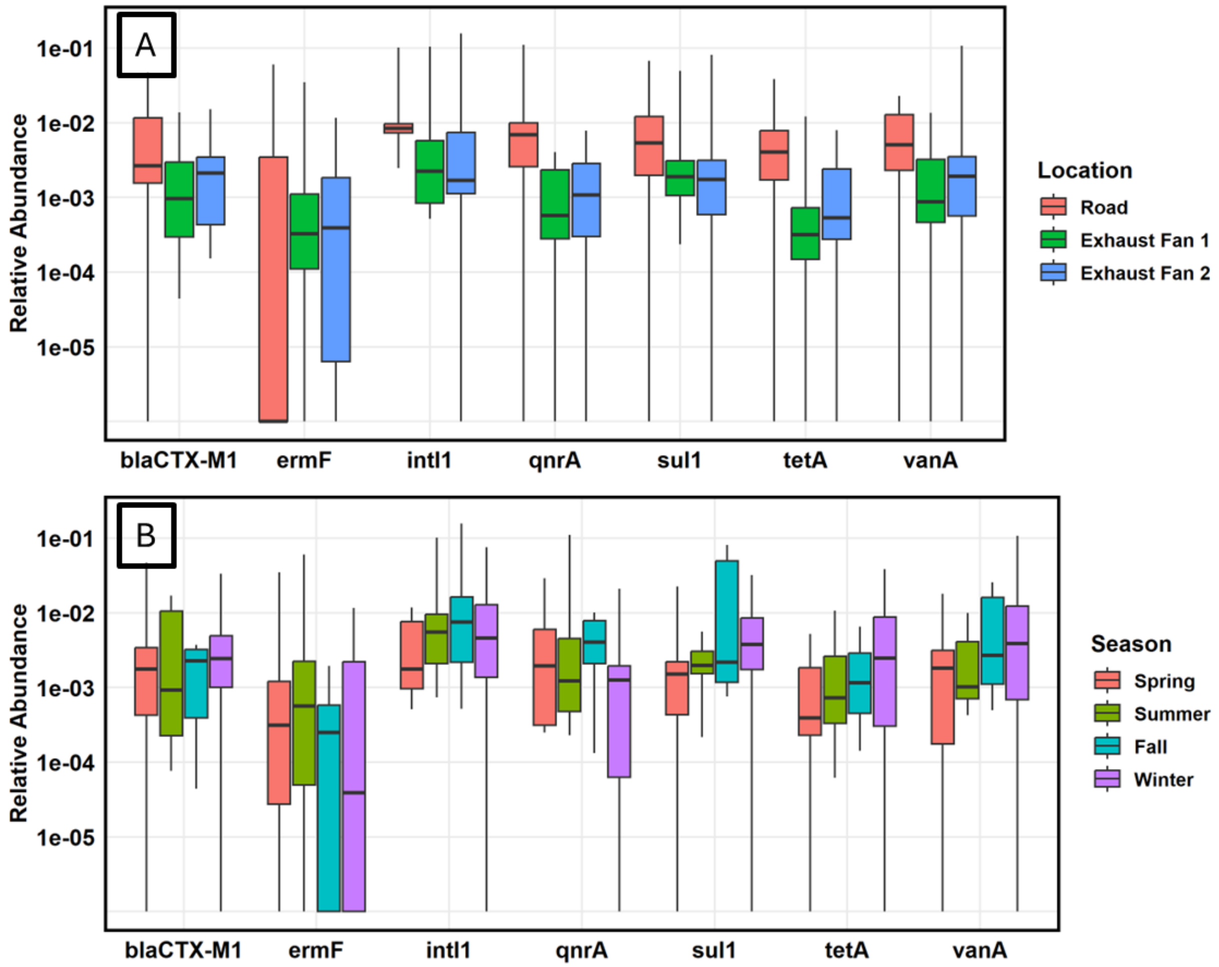

Figure S3. Relative abundance of each target gene (proportion of gene copies of the target to those of the 16S rRNA gene representing total bacteria) in air samples at the swine farm, (A) by location sampled at the swine farm and (B) by the season of the sampling event.

Table S2. Average gene concentrations at exhaust fan 1 (EF1) at the swine farm, sorted by exhaust fan running times.

Location	Exhaust Fan Time (hr)	Gene	Average Conc. (gc/m^3)	Std Dev. (gc/m^3)	Max Conc. (gc/m^3)	Min Conc. (gc/m^3)
EF1	<5	16S rRNA	6.17E+05	8.78E+05	2.15E+06	5.35E+04
EF1	<5	$bla_{CTX\text{-}M1}$	5.78E+02	6.07E+02	1.37E+03	8.57E+01
EF1	<5	*ermF*	1.05E+03	1.92E+03	4.44E+03	0.00E+00
EF1	<5	*intI1*	1.90E+03	2.60E+03	6.46E+03	8.57E+01
EF1	<5	*qnrA*	4.18E+02	4.67E+02	1.03E+03	0.00E+00
EF1	<5	*sul1*	1.09E+03	1.46E+03	3.65E+03	8.57E+01
EF1	<5	*tetA*	5.98E+02	5.40E+02	1.42E+03	0.00E+00
EF1	<5	*vanA*	1.64E+03	2.17E+03	5.32E+03	0.00E+00
EF1	5-20	16S rRNA	4.10E+06	6.37E+06	1.52E+07	3.29E+05
EF1	5-20	$bla_{CTX\text{-}M1}$	2.04E+03	1.57E+03	4.49E+03	6.59E+02
EF1	5-20	*ermF*	3.91E+03	4.44E+03	1.14E+04	3.66E+02
EF1	5-20	*intI1*	2.29E+04	2.85E+04	6.80E+04	4.28E+02
EF1	5-20	*qnrA*	3.53E+03	4.55E+03	1.15E+04	1.71E+02
EF1	5-20	*sul1*	1.46E+04	1.15E+04	3.21E+04	6.93E+02
EF1	5-20	*tetA*	1.14E+03	1.17E+03	2.45E+03	8.57E+01
EF1	5-20	*vanA*	3.06E+03	2.75E+03	7.54E+03	5.59E+02
EF1	>20	16S rRNA	9.16E+07	1.64E+08	3.84E+08	5.39E+06
EF1	>20	$bla_{CTX\text{-}M1}$	1.51E+04	1.14E+04	2.90E+04	2.87E+03
EF1	>20	*ermF*	8.37E+03	8.73E+03	1.89E+04	7.63E+02
EF1	>20	*intI1*	7.25E+04	1.17E+05	2.81E+05	8.82E+03
EF1	>20	*qnrA*	9.16E+04	1.54E+05	3.59E+05	1.49E+03
EF1	>20	*sul1*	4.37E+04	3.63E+04	9.00E+04	6.23E+03
EF1	>20	*tetA*	7.79E+03	9.04E+03	2.35E+04	1.72E+03
EF1	>20	*vanA*	6.36E+04	9.84E+04	2.30E+05	9.49E+02

Table S3. Average gene concentrations at exhaust fan 2 (EF2) at the swine farm, sorted by exhaust fan running times.

Location	Exhaust Fan Time (hr)	Gene	Average Conc. (gc/m^3)	Std Dev. (gc/m^3)	Max Conc. (gc/m^3)	Min Conc. (gc/m^3)
EF2	<5	16S rRNA	3.19E+05	3.13E+05	8.49E+05	4.65E+04
EF2	<5	$bla_{CTX\text{-}M1}$	1.21E+03	2.00E+03	4.78E+03	2.48E+02
EF2	<5	*ermF*	9.01E+02	1.20E+03	2.31E+03	0.00E+00
EF2	<5	*intI1*	1.25E+03	8.92E+02	2.35E+03	3.43E+02
EF2	<5	*qnrA*	6.04E+02	5.99E+02	1.34E+03	8.57E+01
EF2	<5	*sul1*	8.35E+02	1.40E+03	3.29E+03	0.00E+00
EF2	<5	*tetA*	3.84E+02	1.96E+02	6.83E+02	1.71E+02
EF2	<5	*vanA*	2.74E+03	3.67E+03	8.15E+03	0.00E+00
EF2	5-20	16S rRNA	4.12E+06	5.93E+06	1.38E+07	7.45E+04
EF2	5-20	$bla_{CTX\text{-}M1}$	2.30E+03	2.86E+03	6.80E+03	1.71E+02
EF2	5-20	*ermF*	1.80E+03	2.40E+03	5.37E+03	0.00E+00
EF2	5-20	*intI1*	2.16E+04	2.90E+04	6.77E+04	0.00E+00
EF2	5-20	*qnrA*	5.21E+03	9.61E+03	2.23E+04	0.00E+00
EF2	5-20	*sul1*	1.14E+04	1.58E+04	3.71E+04	5.28E+02
EF2	5-20	*tetA*	1.89E+03	3.09E+03	7.31E+03	0.00E+00
EF2	5-20	*vanA*	4.15E+03	4.41E+03	1.02E+04	4.29E+02
EF2	>20	16S rRNA	3.91E+07	4.94E+07	9.54E+07	2.24E+05
EF2	>20	$bla_{CTX\text{-}M1}$	7.44E+03	6.43E+03	1.48E+04	5.94E+02
EF2	>20	*ermF*	2.24E+03	1.87E+03	4.79E+03	4.83E+02
EF2	>20	*intI1*	6.31E+04	8.28E+04	1.62E+05	1.21E+03
EF2	>20	*qnrA*	1.03E+04	1.25E+04	2.68E+04	9.90E+02
EF2	>20	*sul1*	3.63E+04	6.61E+04	1.54E+05	5.11E+02
EF2	>20	*tetA*	8.76E+03	1.33E+04	3.18E+04	5.85E+02
EF2	>20	*vanA*	2.52E+04	3.58E+04	8.15E+04	1.71E+02

Table S4. Average gene concentrations at the road site at the swine farm, sorted by exhaust fan running times, which are the combined times for exhaust fan 1 and exhaust fan 2.

Location	Exhaust Fan Time (hr)	Gene	Average Conc. (gc/m^3)	Std Dev. (gc/m^3)	Max Conc. (gc/m^3)	Min Conc. (gc/m^3)
Road	<12	*16S*	7.17E+04	6.14E+04	1.42E+05	2.22E+04
Road	<12	bla_{CTX-M1}	2.34E+02	2.91E+02	7.39E+02	0.00E+00
Road	<12	*ermF*	1.71E+01	3.83E+01	8.57E+01	0.00E+00
Road	<12	*intI1*	8.27E+02	8.97E+02	2.39E+03	1.71E+02
Road	<12	*qnrA*	3.34E+02	3.10E+02	8.03E+02	0.00E+00
Road	<12	*sul1*	8.60E+02	8.17E+02	1.93E+03	0.00E+00
Road	<12	*tetA*	5.89E+02	4.73E+02	1.22E+03	8.57E+01
Road	<12	*vanA*	7.31E+02	8.78E+02	2.19E+03	0.00E+00
Road	12-24	*16S*	9.84E+04	6.76E+04	2.12E+05	4.21E+04
Road	12-24	bla_{CTX-M1}	1.02E+03	1.39E+03	3.26E+03	8.57E+01
Road	12-24	*ermF*	3.64E+02	4.99E+02	1.19E+03	0.00E+00
Road	12-24	*intI1*	9.45E+03	2.01E+04	4.53E+04	2.57E+02
Road	12-24	*qnrA*	1.16E+03	7.55E+02	2.08E+03	4.25E+02
Road	12-24	*sul1*	2.99E+03	6.26E+03	1.42E+04	0.00E+00
Road	12-24	*tetA*	3.12E+02	4.63E+02	1.13E+03	0.00E+00
Road	12-24	*vanA*	4.73E+02	2.47E+02	7.22E+02	1.71E+02
Road	>24	*16S*	9.19E+04	5.01E+04	1.62E+05	3.15E+04
Road	>24	bla_{CTX-M1}	8.32E+02	1.13E+03	2.75E+03	0.00E+00
Road	>24	*ermF*	2.02E+03	4.29E+03	9.70E+03	0.00E+00
Road	>24	*intI1*	2.70E+03	4.24E+03	1.02E+04	2.57E+02
Road	>24	*qnrA*	2.44E+03	4.86E+03	1.11E+04	8.57E+01
Road	>24	*sul1*	4.72E+02	2.02E+02	7.07E+02	2.23E+02
Road	>24	*tetA*	3.78E+02	3.26E+02	9.50E+02	1.71E+02
Road	>24	*vanA*	5.40E+02	3.90E+02	1.00E+03	8.57E+01

Text S2. Description of size distributions at the road site.

At the road site, the fraction of *intI1* and *sul1* in accumulation mode particles was significantly higher than in coarse particles and fine particles respectively, with a maximum of 0.55 for *sul1*. This suggests that *intI1* and *sul1* may be more associated with smaller particles that can travel further from the source, indicating a potential outside source for these genes. This finding agrees well with the significant correlation between these two genes and wind speed. As at the dairy farm,

coarse particles carried a larger fraction of the targeted genes at sampling sites close to the source (i.e., the two exhaust fans compared to the road site).

Text S3. Evaluation of uncertainty in emission rates derived from Gaussian dispersion modeling. In our evaluation of the emission rates for the three genes of interest, the results were mixed but showed some notable trends, particularly for $bla_{CTX\text{-}M1}$. We employed a bootstrapping approach to evaluate the uncertainty in the emission rates. After calculating the emission rates as described, we incorporated them back into the Gaussian dispersion model to solve for a predicted concentration for each sample and compare it to the observed concentration. This was done using the wind speed data collected from an anemometer and vane from a single sampler. For the 16S rRNA gene, 11 out of 18 calculated emission rates were greater than zero. At the 65-m location, the mean average percent error of the concentrations predicted using these 11 emission rates was 32%. However, at the 115-m location, only three of the 11 predicted concentrations were of the same order of magnitude as the observations, with the majority showing significant variation due to observed concentrations being only slightly above the background or upwind measurements, which were typically near the detection limit of the method. Thus, comparisons between the bootstrapped and observed concentrations were difficult to interpret at this location. The mean average percent error for the three of the 115-m samples above background was 47%, reflecting the increased challenge at this distance.

For $bla_{CTX\text{-}M1}$, 12 of 18 predicted emission rates were greater than zero, and the results were more consistent overall. At the 65-m site, 10 of the 12 predicted concentrations were within the same order of magnitude as their measured counterparts. The mean average percent error associated with 12 samples at 65 m was 32%. At 115 m, the results were somewhat better than with the 16S rRNA

gene, with predicted concentrations for seven of the 12 samples falling within the same order magnitude as observations. The mean average percent error for these seven samples was 53%. However, five samples produced concentrations that were undetectable. For three of these, the calculated concentrations were very low or close to zero, aligning with expectations. For the remaining two samples, the bootstrapped concentrations were much higher, pointing to inconsistencies in those measurements.

For *intI1*, 10 of 18 predicted emission rates were greater than zero. At the 65-m location, nine out of 10 calculated concentrations were within the same or of magnitude as the observations, while at the 115-m location, the same was true for five out of 10 samples. The mean average percent error for all 10 samples at the 65-m location was 28%, and for the seven samples above background at the 115-m location, it was 59%. Similar to 16S rRNA, the measured concentrations for the other three samples were close to background or undetectable at 115 m. Again, small differences between the upwind/background location and the 115-m downwind point contributed to the discrepancies. Overall, the Gaussian plume dispersion model most consistently reflected observations for $bla_{CTX\text{-}M1}$, which corresponds to the antibiotic used at the farm and supports the barn as a direct, measurable source of this gene, with 16S rRNA and *intI1* involving some challenges at longer distances due to their low concentrations.

Text S4. Evaluation of exposure to ARGs.

Exposure was calculated using factors listed in the US Environmental Protection Agency (EPA) Exposure Factors Handbook. These factors are essential for translating environmental contaminant concentrations into estimated human exposure levels across different pathways. Exposure was

calculated by multiplying the concentrations of ARGs measured in air and soil at specific sampling locations by the intake rates for adults (>21 years old), representative of the workers at the farms, of air (20 m^3 d^{-1}) and soil (50 mg d^{-1}).[11] This provided an estimate of the total daily exposure at various locations on the dairy farm.

Predicted exposure to ARGs differed significantly between inhalation and ingestion pathways. Figure S6 highlights the exposure rates for the inhalation of aerosols 5 m from the pen and ingestion of the pen soil, and inhalation of aerosols downwind compared to ingestion of soil respectively, representing cases where workers are near or inside the special-needs barn and when workers are further downwind of the special-needs barn. For the former case closer to the special-needs barn, $bla_{CTX\text{-}M1}$ exposure would be significantly higher from inhalation compared to ingestion, while the opposite was true for 16S rRNA, *ermF, qnrA*, *sul1, tetA*, *vanA*, and *intI1*. For the latter condition downwind of the special-needs barn, $bla_{CTX\text{-}M1}$, *ermF*, and *sul1* exposure would be comparable or significantly higher for *ermF* from inhalation compared to ingestion. For total bacteria in terms of 16S rRNA, *qnrA*, *tetA*, *vanA*, and *intI1,* exposure would be significantly higher from ingestion of soil compared to downwind inhalation. Among the target genes, *intI1* produced the highest levels of exposure, reaching ~10^6 gc d^{-1}, similar to the exposure rates in fecal matter reported in previous studies.[12]

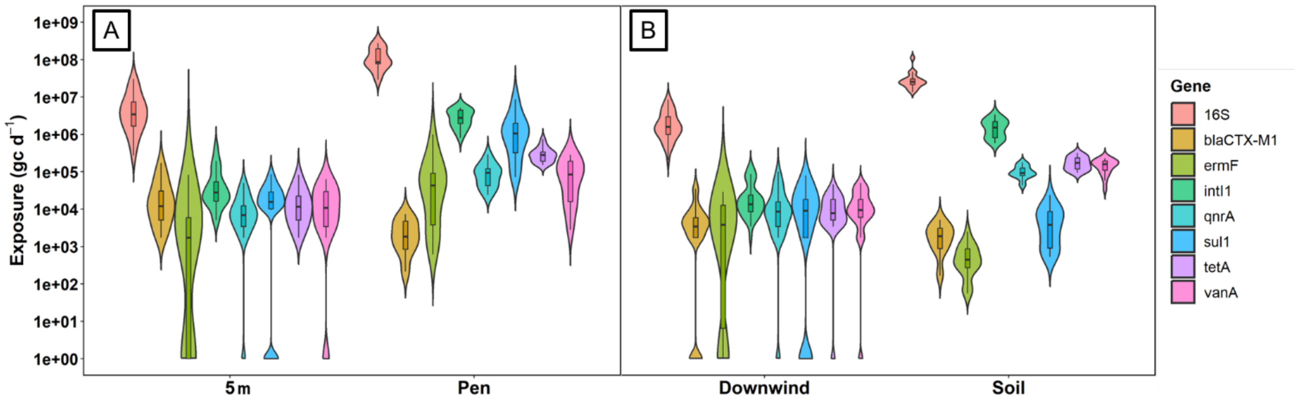

Figure S4. Estimated exposure rates to target genes at the dairy farm by (a) inhalation of aerosols near the special-needs barn compared to ingestion of manure from the pens in the special-needs barn, and (b) inhalation of aerosols tens of meters downwind from the special-needs barn compared to ingestion of soil downwind.

References

(1) Wark, K.; Warner, C. F.; Davis, W. T. *Air Pollution. Its Origin and Control*, 3rd ed.; Addison-Wesley: Menlo Park, 1998. https://doi.org/10.1016/0013-9327(77)90079-9.

(2) Jahne, M. A.; Rogers, S. W.; Holsen, T. M.; Grimberg, S. J. Quantitative Microbial Risk Assessment of Bioaerosols from a Manure Application Site. *Aerobiologia (Bologna)* **2015**, *31* (1), 73–87. https://doi.org/10.1007/s10453-014-9348-0.

(3) Suzuki, M. T.; Taylor, L. T.; DeLong, E. F. Quantitative Analysis of Small-Subunit RRNA Genes in Mixed Microbial Populations via 5'-Nuclease Assays. *Appl Environ Microbiol* **2000**, *66* (11), 4605–4614. https://doi.org/10.1128/AEM.66.11.4605-4614.2000.

(4) Kim, J.; Lim, Y. M.; Jeong, Y. S.; Seol, S. Y. Occurrence of CTX-M-3, CTX-M-15, CTX-M-14, and CTX-M-9 Extended-Spectrum β-Lactamases in Enterobacteriaceae Clinical Isolates in Korea. *Antimicrob Agents Chemother* **2005**, *49* (4). https://doi.org/10.1128/AAC.49.4.1572-1575.2005.

(5) Chen, J.; Yu, Z.; Michel, F. C.; Wittum, T.; Morrison, M. Development and Application of Real-Time PCR Assays for Quantification of Erm Genes Conferring Resistance to Macrolides-Lincosamides-Streptogramin B in Livestock Manure and Manure Management Systems. *Appl Environ Microbiol* **2007**, *73* (14), 4407–4416. https://doi.org/10.1128/AEM.02799-06.

(6) Goldstein, C.; Lee, M. D.; Sanchez, S.; Hudson, C.; Phillips, B.; Register, B.; Grady, M.; Liebert, C.; Summers, A. O.; White, D. G.; Maurer, J. J. Incidence of Class 1 and 2 Integrases in Clinical and Commensal Bacteria from Livestock, Companion Animals, and Exotics. *Antimicrob Agents Chemother* **2001**, *45* (3). https://doi.org/10.1128/AAC.45.3.723-726.2001.

(7) Cattoir, V.; Poirel, L.; Rotimi, V.; Soussy, C. J.; Nordmann, P. Multiplex PCR for Detection of Plasmid-Mediated Quinolone Resistance Qnr Genes in ESBL-Producing Enterobacterial Isolates. *Journal of Antimicrobial Chemotherapy* **2007**, *60* (2). https://doi.org/10.1093/jac/dkm204.

(8) Pei, R.; Kim, S. C.; Carlson, K. H.; Pruden, A. Effect of River Landscape on the Sediment Concentrations of Antibiotics and Corresponding Antibiotic Resistance Genes (ARG). *Water Res* **2006**, *40* (12), 2427–2435. https://doi.org/10.1016/j.watres.2006.04.017.

(9) Ng, L. K.; Martin, I.; Alfa, M.; Mulvey, M. Multiplex PCR for the Detection of Tetracycline Resistant Genes. *Mol Cell Probes* **2001**, *15* (4). https://doi.org/10.1006/mcpr.2001.0363.

(10) Dutka-Malen, S.; Evers, S.; Courvalin, P. Detection of Glycopeptide Resistance Genotypes and Identification to the Species Level of Clinically Relevant Enterococci by PCR. *J Clin Microbiol* **1995**, *33* (1), 24–27. https://doi.org/10.1128/jcm.33.1.24-27.1995.

(11) US EPA (United States Environmental Protection Agency) Washington, D. Exposure Factors Handbook: 2011 Edition Office of Research and Development, United States Environmental Protection Agency. **2011**.

(12) Wang, Y.; Li, Y.; Li, H.; Zhou, J.; Wang, T. Seasonal Dissemination of Antibiotic Resistome from Livestock Farms to Surrounding Soil and Air: Bacterial Hosts and Risks for Human Exposure. *J Environ Manage* **2023**, *325*. https://doi.org/10.1016/j.jenvman.2022.116638.
